# Materials, Devices, and Systems of On‐Skin Electrodes for Electrophysiological Monitoring and Human–Machine Interfaces

**DOI:** 10.1002/advs.202001938

**Published:** 2020-12-04

**Authors:** Hao Wu, Ganguang Yang, Kanhao Zhu, Shaoyu Liu, Wei Guo, Zhuo Jiang, Zhuo Li

**Affiliations:** ^1^ Flexible Electronics Research Center State Key Laboratory of Digital Manufacturing Equipment and Technology School of Mechanical Science and Engineering Huazhong University of Science and Technology Wuhan Hubei 430074 China; ^2^ Department of Materials Science Fudan University Shanghai 200433 China

**Keywords:** electrophysiological monitoring, human–machine interfaces, on‐skin electrodes, stretchable electronics

## Abstract

On‐skin electrodes function as an ideal platform for collecting high‐quality electrophysiological (EP) signals due to their unique characteristics, such as stretchability, conformal interfaces with skin, biocompatibility, and wearable comfort. The past decade has witnessed great advancements in performance optimization and function extension of on‐skin electrodes. With continuous development and great promise for practical applications, on‐skin electrodes are playing an increasingly important role in EP monitoring and human–machine interfaces (HMI). In this review, the latest progress in the development of on‐skin electrodes and their integrated system is summarized. Desirable features of on‐skin electrodes are briefly discussed from the perspective of performances. Then, recent advances in the development of electrode materials, followed by the analysis of strategies and methods to enhance adhesion and breathability of on‐skin electrodes are examined. In addition, representative integrated electrode systems and practical applications of on‐skin electrodes in healthcare monitoring and HMI are introduced in detail. It is concluded with the discussion of key challenges and opportunities for on‐skin electrodes and their integrated systems.

## Graphical abstract

On‐skin electrodes function as an ideal platform for collecting high‐quality electrophysiological signals due to their unique characteristics. This review summarizes the latest progress of on‐skin electrodes, with particular interest in advanced materials for the fabrication, adhesion, and breathability of electrodes, as well as their wearable applications. Future challenges and opportunities for on‐skin electrodes are also discussed.

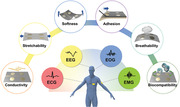

## 1 Introduction

Most human body movements are driven by low‐level electrical potentials referred to as electrophysiological (EP) signals, including electrocardiogram (ECG),^[^ ^1^, ^2^ ^]^ electrooculogram (EOG),^[^ ^3^ ^]^ electromyogram (EMG),^[^ ^4^, ^5^ ^]^ and electroencephalogram (EEG).^[^ ^6^, ^7^ ^]^ Among them, ECG is directly correlated to myocardial conduction, thus is usually employed as a metric to detect cardiovascular diseases, such as arrhythmia.^[^ ^8^ ^]^ EOG signals stem from the potential difference created by the eye as a dipole via a positively charged cornea and a negatively charged retina. They enable the feedback of eye movements or retinal stimulation, paving the way for mobile eye therapies and eye‐movement control.^[^ ^9^, ^10^ ^]^ As an index of the initiation and fatigue of activated muscles, EMG signals originate from electrical activities produced by muscle of interest and are suitable for monitoring muscular diseases and mental fatigue etc.^[^ ^11^ ^]^ Particularly, the characteristics of EMG signals from muscles on forearm can be directly correlated to hand gestures,^[^ ^12^ ^]^ therefore, EMG signals are widely used for gesture recognition,^[^ ^13^ ^]^ prosthetic control,^[^ ^14^ ^]^ etc. EEG signals obtained from human scalp surface capture the post‐synaptic potentials generated by pyramidal neurons,^[^ ^15^ ^]^ thus can be utilized to assist brain‐activity cognition and mental disease diagnosis, such as epilepsy diagnoses.^[^ ^16^ ^]^ The change in brain patterns as a response to voluntary or involuntary mental command can be reflected by EEG signals, which can be applied as a feasible method for brain–computer interface (BCI).^[^ ^17^ ^]^ Due to the rich information extracted from them, EP signals have inspired great research interest and they have been widely used in healthcare monitoring and HMI.

Electrodes are essential components to enable the capture and analysis of EP signals. However, conventional Ag/AgCl electrodes have plenty of limitations. For instance, the gel used in commercial electrodes to reduce contact impedance may cause skin irritation after long term measurements. In addition, the rigid nature of the Ag/AgCl electrodes hinders them from collecting high‐quality signals during motion due to the loss of contact with skin.^[^ ^18^ ^]^ With the recent rise of flexible electronics, on‐skin electrodes have received tremendous attention in the past few years.^[^ ^19^, ^20^, ^21^, ^22^, ^23^ ^]^ With continuous optimizations, on‐skin electrodes become promising alternatives to Ag/AgCl electrodes. To be specific, on‐skin electrodes have achieved excellent stretchability of over 400%, enough to ensure high fidelity measurements during large degree of human motion. By virtue of skin‐matched modulus and ultralow thickness,^[^ ^24^, ^25^ ^]^ on‐skin electrodes are capable of providing compliant and comfortable interfaces with skin, reducing measurement impedance, and increasing signal‐to‐noise ratio (SNR).^[^ ^26^, ^27^ ^]^ The elimination of electrode gel also enhances wearable comfort and reduce the risk of skin irritation. Through integration with power and wireless communication modules to form standalone systems, emerging on‐skin electrodes exhibit remarkable features in mobile and wearable applications for personalized healthcare and HMI.^[^ ^28^, ^29^ ^]^

This review focuses on latest advancements of on‐skin electrodes, with particular interest in advanced materials for the fabrication, adhesion and breathability of electrodes, as well as their wearable applications. We will first analyze desirable properties of on‐skin electrodes, followed by the research progress achieved so far toward those features. Particularly, we start with in‐depth analysis of recent progress in electrode material synthesis and optimization, and the discussion on strategies to enhance adhesion and breathability of on‐skin electrodes will be presented afterward. Furthermore, emerging integrated electrode systems and wearable applications of on‐skin electrodes will be introduced. We conclude this article with a summary on past progress and perspectives for challenges and opportunities of on‐skin electrodes.

## 2 Desirable Properties of On‐Skin Electrodes

**Figure 1:**
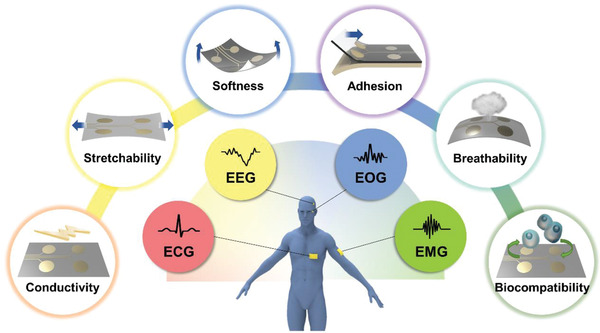
A schematic illustration of desirable properties of on‐skin electrodes.

Ideal on‐skin electrodes are supposed to possess important features in order to continuously obtain high‐fidelity EP signals with wearable comfort. As shown in **Figure** **1**, those essential features include conductivity, stretchability, softness, adhesion to skin, breathability, and biocompatibility. It is noted that the capability of self‐healing has also attracted significant attention, and impressive results have been reported.^[^ ^30^, ^31^ ^]^ Since there are already reviews dedicated to self‐healing electronics,^[^ ^32^ ^]^ this review will focus on the fundamental features mentioned above which enable the functionality of on‐skin electrodes.

There are plenty of noisy interferences on the skin–electrode interfaces, which are likely to reduce the quality of signals recorded by electrodes.^[^ ^33^ ^]^ Due to high charge carrier density and surface electric displacement field of highly conductive materials, electrodes with remarkable conductivity will be beneficial for enhancing the SNR of measurements. Thus, improving the conductivity of the electrode is of great importance. Conventional rigid metal electrodes (Ag/AgCl or stainless steel electrodes) have excellent conductivity but cannot fulfill wearable function, while flexible or stretchable electrodes typically exhibit relatively lower conductivity. Therefore, it is imperative to develop highly conductive electrode materials for the fabrication of flexible or stretchable on‐skin electrodes.

Stretchability, namely, the capability to accommodate large degree of deformation, is another critical factor for on‐skin electrodes. Mechanical strain exerted on the device during external loading conditions, such as bending, stretching, twisting, etc., can well exceed the fracture strain of the electrode materials (≈1% for metallic materials and ≈5% for conductive polymer).^[^ ^34^, ^35^ ^]^ Meanwhile, human skin can survive mechanical strain of ≈15% without irreversible damage,^[^ ^36^ ^]^ on‐skin electrodes are required to possess matched mechanical properties with human skin. In fact, the stretchability of human skin can be fairly large depending on the locations and loading conditions, e.g., the failure strain of human back skin ranges from 37% to 71% during quasistatic tests, while that of forehead and arm ranges from 27% to 59% under dynamic load.^[^ ^37^ ^]^ Therefore, with the linear elastic limit of the skin as a reference,^[^ ^38^ ^]^ the on‐skin electrodes are also required to have high stretchability (at least >15%) to maintain conformal contact with skin and ensure stable monitoring during skin deformation.

Softness of electrodes, as a different physical property from stretchability, is also critically important for on‐skin electrodes. Softness of the electrodes can prevent skin/tissue from damage and contribute to wearable comfort. Softness is typically defined by hardness, which is evaluated through the indentation test in mechanics. In the meantime, softness is closely related to the Young's modulus of the material.^[^ ^39^ ^]^ Therefore, the softness of on‐skin electrodes is also evaluated through the comparison with the modulus of human skin/tissue (0.5–1.95 MPa^[^ ^40^ ^]^) . Needless to say, owing to the low Young's modulus of human skin, on‐skin electrodes are required to exhibit similar modulus to ensure compliance with skin to avoid damage.

Adhesion to skin is critical since robust contact between the electrodes and skin mainly depends on strong interface adhesion.^[^ ^41^, ^42^ ^]^ When skin is severely deformed or the electrode is laminated on sweaty skin surface, it may fail to record EP signals due to delamination. Moreover, sufficient adhesion to the skin reduces the interface impedance of the skin‐mountable electrode, ensuring high SNR and accuracy.

Medical research indicates that about 500 mL water is evaporated from human skin every day.^[^ ^43^ ^]^ Therefore, breathability is an essential consideration for long‐term usability of on‐skin electrodes, as a stagnant water layer between the electrode and skin produces signal drift and even causes skin allergies and inflammation.^[^ ^44^ ^]^ However, most on‐skin electronics developed so far were made of materials with limited breathability, which constrained sweat evaporation and gas/vapor diffusion.

In addition to the features mentioned above, biocompatibility of the electrodes also plays a crucial role. Studies have demonstrated that the conductive gel of commercial Ag/AgCl electrodes will cause skin irritation or inflammation when used for a long time.^[^ ^45^ ^]^ On‐skin electrodes usually take the form of dry electrode, i.e., without the adoption of electrode gel. However, the direct contact between electrodes and skin may also cause health concern. Therefore, the development of biocompatible on‐skin electrodes has great significance for nonirritating long‐term EP monitoring.^[^ ^46^, ^47^, ^48^ ^]^

## 3 Materials for On‐Skin Electrodes

As discussed above, mechanical and electrical properties of the electrode materials have great impact on the measurement performances of on‐skin electrodes, and a great deal of research efforts have been devoted to the synthesis and fabrication of high‐performance electrode materials. In this section, we will summarize recent research advances in on‐skin electrodes based on material innovations, particularly electrodes based on metal thin film, carbon materials, metallic nanomaterials, and other materials such as conductive polymers and hydrogel, etc.

### 3.1 Metal Thin Film Electrodes

**Figure 2:**
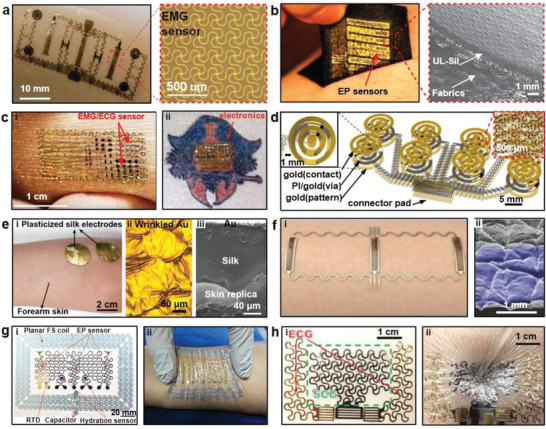
Metal thin film electrodes. a) On‐skin electrodes with serpentine design. Reproduced with permission.^[^ ^55^ ^]^ Copyright 2016, Wiley‐VCH. b) Stretchable electrodes with UL‐Sil and fabrics. Reproduced with permission.^[^ ^56^ ^]^ Copyright 2014, Springer Nature. c‐i) Epidermal electrode systems incorporating EMG/ECG sensors and ii) image of the on‐skin electronics integrated onto a tattoo. Reproduced with permission.^[^ ^57^ ^]^ Copyright 2011, AAAS. d) A schematic of TCR electrodes and the magnified views of Peano curves (inset). Reproduced with permission.^[^ ^58^ ^]^ Copyright 2015, National Academy of Sciences USA. e) Photographs of gold film electrodes with wrinkled structures on the plasticized silk substrate. Reproduced with permission.^[^ ^60^ ^]^ Copyright 2018, Wiley‐VCH. f‐i) An illustration of optimized on‐skin electrodes and ii) an SEM image shows the superior conformality of a 5 µm thick membrane. Reproduced with permission.^[^ ^61^ ^]^ Copyright 2013, Wiley‐VCH. g‐i) Multifunctional device consisting of EP sensors and ii) an image shows conformal skin‐electrode interfaces during compression. Reproduced with permission.^[^ ^66^ ^]^ Copyright 2015, Wiley‐VCH. h) Images of the SCG and ECG sensors with excellent conformal contact with skin. Reproduced with permission.^[^ ^67^ ^]^ Copyright 2019, Wiley‐VCH.

Metals have superior conductivity and mechanical properties, thus they are widely used as electrode materials.^[^ ^49^, ^50^, ^51^ ^]^ In addition, metal electrodes can be manufactured through state‐of‐the‐art micro/nanofabrication technologies such as magnetron sputtering,^[^ ^52^ ^]^ electrochemical deposition,^[^ ^53^ ^]^ etc., which are scalable with relatively low costs. However, bulk metals typically have very low breakage strain and large modulus.^[^ ^54^ ^]^ To enhance the flexibility and stretchability of electrodes, metals are usually fabricated in the form of thin‐film on stretchable substrates with serpentine or meander patterns. Xu et al. presented conformal on‐skin electrodes using metal thin films for EMG signal acquisition (**Figure** **2**a).^[^ ^55^ ^]^ The electrodes were composed of a sputtered bilayer of Au/Cr (5 nm/200 nm) and silicone substrate (Young's modulus of 60 kPa). The overall device demonstrated Young's modulus of about 69 kPa with the thickness of only ≈20 µm. With serpentine geometry, the maximum strain on the metal electrode was less than 0.3% when the device was subject to 21% tensile deformation. Jang and co‐workers adopted a composite substrate made of an ultralow modulus silicone (UL‐Sil) and elastic fabric to enhance the softness, stretchability, adhesion, and breathability of the thin‐film metal electrodes (Figure 2b).^[^ ^56^ ^]^ The ultrathin UL‐Sil (Young's modulus of ≈3 kPa, thickness ≈2 µm) offered a highly compliant and adhesive surface integrated with skin to achieve conformal contact, even in areas with dense hair. And the fabric was employed as a reliable strain limiting support platform for the UL‐Sil to improve mechanical integrity and stretchability of this device. As a result, the Au/Cr film electrode demonstrated excellent stretchability, i.e., the device did not fracture until the tensile strain reached 220%.

In addition to the use of low modulus elastomer substrate to promote the softness and stretchability of metal film electrodes, structural designs were also proved to be effective. Kim et al. designed tattoo‐like epidermal electronic systems (Figure 2c), where the electrodes were in the form of filamentary serpentine (FS) nanoribbons structures.^[^ ^57^ ^]^ With the microstructure design, the epidermal electronic system achieved effective elastic moduli of ≈140 kPa, bending stiffness of <1 nN m^−1^. Besides, the stress‐strain curve of loading and unloading showed a purely elastic response when the loading strain reached 30%, at which point the maximum principal strain was less than ≈0.2% in the metal film. A new type of self‐similar structure was proposed by Norton et al.^[^ ^58^ ^]^ The device adopted a specially designed tripolar concentric ring (TCR) geometry of soft, collapsible electrodes that closely conformed to complex surface topologies of the auricle and mastoid. The active portion of the TCR electrode was arranged in a spatially varying and self‐similar design using a Peano curve as the building unit, as shown in Figure 2d. It was noted that the geometric features were tailored with orientational anisotropies such that vertical Peano curves were adopted to maximize stretchability along longitudinal axes for interconnects and a half‐and‐half designed was used for electrodes to balance stretchability in all directions. Due to the structural design, the thin film electrodes had excellent characteristics of stretchability (>50%) and bendability (>180°). Moreover, Kim et al. proposed compliant interconnect designs without additional backing layers for the Au/Ti electrode array.^[^ ^59^ ^]^ Without the restriction of supporting layers, the compliant interconnect could move freely. When the electrode array was applied on human skin, the compliant interconnects absorbed the deformation of the electrode to achieve conformal contact. Tensile tests verified that the compliant interconnects improved electrode stretchability effectively. And the electrode array could be stretched by 55% or compressed by 30% without any damage. Since both soft substrate and structure design are beneficial for improved mechanical properties, Chen and co‐workers improved the softness and stretchability of metal‐film electrodes through integrating wrinkled structures on soft silk protein substrate (Figure 2e).^[^ ^60^ ^]^ With the addition of 30 wt% CaCl_2_ and moisture from ambient environment, the original tough silk protein was plasticized and the Young's modulus was reduced from 5–12 GPa to 0.1–2 MPa. Subsequently, Au films were deposited on plasticized silk and formed wrinkled structures simultaneously due to the ambient hydration. The device stretchability was notably increased from 20% to 400%. Additionally, the relative resistance change was only ≈2.45 under 40% strain.

To investigate the factors affecting the performances of EMG signals measured by metal film electrodes, Jeong et al. carried out systematic studies by changing the thickness, shape, size, and spacing of the electrodes and determined the optimized metal thin‐film electrodes (Figure 2f).^[^ ^61^ ^]^ During mechanics analysis and experiment verification, it was shown that there was a critical thickness of the device to maintain conformal contact with the skin (i.e., thickness: 25 µm). Due to the excellent conformality (Figure 2f), this study adopted the prototype with 5 µm thick membranes. In addition, the conformal contact also led to an increased SNR. Two electrode geometry, bar type, and circular type, and interelectrode distance of 10, 20, 30, 40, and 60 mm, were evaluated. It was found that the bar type electrode increased the possibility of signal detection due to the alignment of the electrode with muscle fibers. The optimum inter‐electrode distance was determined to be 20 mm with minimum crosstalk from muscles.

It has been shown that metal film electrodes are likely to cause skin irritation and allergies after directly exposed to the skin for a long time.^[^ ^62^ ^]^ In addition to preparing metal film electrodes with biocompatible metal materials, such as Au, another appropriate approach to mitigate the biocompatible risk is to adopt capacitive electrodes, which eliminates direct contact to the skin by encapsulating metal electrodes with biocompatible substrate. In addition, there was no direct electrode contact or charge transport in this capacitive sensing mode, which eliminated the leakage between electronic components. Lee et al. developed capacitive electrodes composed of metal thin film sandwiched between two PDMS layers.^[^ ^63^ ^]^ The metal electrode layer consisted of 30 nm Ti, 10 µm Cu, 30 µm Ni, and 100 nm Au, and the biocompatible PDMS layers in contact with skin ensured that no skin irritation such as itching or erythema occurred. Despite the protection of skin from direct contact with the metal layer, the large height of the device (4 mm) led to the difficulty in forming conformal contact with skin and discomfort. To maintain intimate contact with skin during movement, Jeong et al. designed an ultrathin capacitive gold electrode (200 nm in thickness) with a silicone insulation layer.^[^ ^64^ ^]^ The thin, stretchable electrode effectively realized conformal contact with skin in real‐time through van der Waals interaction. The measurement validated that when 5 mA current flowed through the electrode, the leakage currents of capacitive electrodes were less than 10 nA in both dry and wet conditions to protect the skin from electrical irritation.

Although the aforementioned metal thin film electrodes were able to record signals effectively, the manufacturing processes require photolithography, high temperature, and a high level of vacuum environment.^[^ ^65^ ^]^ Therefore, it is of great value to develop low‐cost and facile manufacturing processes for metal thin film electrodes. Yang et al. manufactured on‐skin devices by using a “cut‐and‐paste” method, which was able to replace standard photolithograph.^[^ ^66^ ^]^ Among them, the device was composed of the EP patch and a hydration sensor, enabling the conformal contact during deformation conditions (Figure 2g). And the designed electrode patterns were directly carved out by a programmable cutting machine on thin metal films. Compared with photolithography, it took less than 10 min to complete the prototype by printing the circuit patterns onto adhesives and tapes. Moreover, the “cut‐and‐paste” method was compatible with roll‐to‐roll manufacture scheme, potentially enabling high volume production of on‐skin electrodes. The same group also extended the manufacturing scheme to fabricate a multifunctional sensor with excellent conformality that could measure both ECG signals and Seismocardiography (SCG), as shown in Figure 2h.^[^ ^67^ ^]^ The vibration of the chest was measured by cut‐and‐pasted polyvinylidene fluoride (PVDF) thin film. And the cut‐and‐paste fabrication scheme was proved to be effective as the device could accommodate a high strain of over 110% while achieving a sensitivity of 0.4 mV per microstrain (*µε*). Moreover, the device maintained normal performances after 10 000 stretching cycles when applied strain was as large as 20%. Guo et al. reported a liquid metal spray‐painting method to pattern an inter‐digital array (IDA) electrode directly on skin.^[^ ^68^ ^]^ GaIn24.5 was used as the conductive ink, which was biocompatible and of low costs. When fabricated on skin through spray‐painting, the liquid metal electrode pattern was deposited on the predesigned stainless mask by chemical etching. And the GaIn24.5 rapidly formed a thin oxide film to maintain the printed patterns at room temperature. The spray‐painting approach achieved a relatively high line width resolution of 100 µm. And the IDA electrode was adopted to test impedance spectroscopy on pigskin, indicating potential applications for EP signal monitoring.

### 3.2 Carbon Material Electrodes

**Figure 3:**
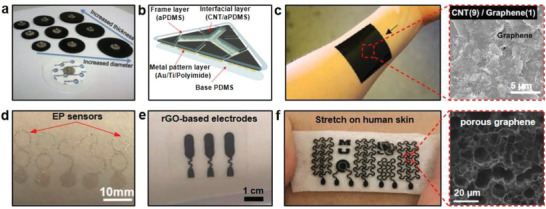
Carbon material electrodes. a) Images of nine CNT/PDMS electrodes. Reproduced with permission.^[^ ^72^ ^]^ Copyright 2012, IEEE. b) A schematic illustration of a CNTs/aPDMS ECG electrode. Reproduced with permission.^[^ ^75^ ^]^ Copyright 2014, Springer Nature. c) The CNT/graphene electrode patch with the content of CNT and graphene at 9:1. Reproduced with permission.^[^ ^76^ ^]^ Copyright 2016, American Chemical Society. d) A photograph of the graphene electronic tattoo containing EP sensors. Reproduced with permission.^[^ ^82^ ^]^ Copyright 2017, American Chemical Society. e) rGO‐aPDMS electrodes for monitoring EP signals. Reproduced with permission.^[^ ^85^ ^]^ Copyright 2020, Elsevier. f) EP electrodes with laser‐induced porous graphene. Reproduced with permission.^[^ ^86^ ^]^ Copyright 2018, Wiley‐VCH.

Carbon nanotubes (CNTs) have played a critical role in the development of on‐skin electrodes due to their excellent characteristics, such as flexibility and high electrical conductivity.^[^ ^69^, ^70^, ^71^ ^]^ A straightforward strategy to fabricate CNTs based electrodes was to mix CNTs with silicone substrate to achieve a balance of conductivity and stretchability.^[^ ^71^ ^]^ However, owing to strong van der Waals interaction between CNTs, mixing the CNTs in silicone evenly is challenging. Jung et al. developed a CNT/PDMS electrode in which CNTs in PDMS were dispersed efficiently by a three‐roll milling machine.^[^ ^72^ ^]^ The dispersion process required only two steps. Highly concentrated CNT was first dispersed under high shear flow condition, followed by dilution to required concentration. The results of nine electrodes (**Figure** **3**a) of different testing conditions showed that the 4.5wt% CNT/PDMS composite electrodes maintained almost constant electrical conductivity of ≈10 S cm^−1^, even when the tensile strain was varied from 5% to 45%. In addition, there was no skin irritation even after continuously wearing the electrodes for 7 days. Another straightforward dispersion approach was explored by Liu et al.^[^ ^73^ ^]^ The method consisted of three steps without requiring expensive instruments. The CNTs were firstly dispersed in solvents by adopting the ultrasonication method. Afterward, PDMS monomer was added to the CNT suspension and dispersed again by stirring. Lastly, mixing the hardening agent into the CNT/PDMS composite achieved the in situ polymerization to yield homogenous dispersion. It was demonstrated the signal amplitude of the 5 wt% CNT content electrodes fabricated by 12 h of ultrasonication was comparable to that from commercial Ag/AgCl electrodes. The same group optimized the above method to obtain conductive composites of higher CNT density.^[^ ^74^ ^]^ A heat‐stirring process was adopted to avoid particle agglomeration. And the content of CNTs was increased to 11 wt% for high conductivity. In order to enhance the electrode flexibility, silver nanoparticles were added to reduce overall stiffness.

Another approach to obtain even dispersion is to create additional wetting of CNTs, the surface energy of the CNTs decreases as a result, leading to easier dispersion of CNTs in a viscous fluid. Lee et al. adopted a solvent‐wetting approach to mix CNTs in adhesive PDMS (aPDMS).^[^ ^75^ ^]^ Ethanol was utilized to wet CNTs before the aPDMS was added. Then, the composite was stirred for 15h under intensive shear flow. And the results showed that the CNTs were homogeneously diffused into the mixture with no empty spaces. Based on the CNTs/aPDMS composite, they designed a self‐adhesive, highly conformal triangle electrode. As shown in Figure 3b, the patch was composed of PDMS substrate, an Au/Ti/Polyimide (PI) metal layer, an aPDMS frame layer, and a CNT/aPDMS interfacial layer for recording the signal. As the metal layer consisted of 1 mm^2^ small pieces connected with strain‐relief serpentine structure, the electrode could survive 30% tensile strain without mechanical damage. Due to the addition of aPDMS, the thin (120 µm) composite electrode had low modulus (27.5 kPa), which was sufficient for conformal contact to improve the measurement fidelity. The electrodes also demonstrated excellent biocompatibility as no itching or erythema occurred after laminated onto skin for a week. Different from previously mentioned approaches, Kim et al. enhanced the stretchability and conductivity of CNT‐based electrodes by preparing a CNT/Graphene filled nanocomposite.^[^ ^76^ ^]^ Among them, the composite was composed of CNTs, second filler (carbon black, nanostructured graphite, and graphene nanopowder), and PDMS. The studies showed that when the composite electrodes were synthesized by mixing graphene with CNT at an optimized filler ratio (1:9, total filler content: 1 wt%), as shown in Figure 3c, they had minimal volume resistance of ≈100 Ω cm, which was 33% increase in conductivity compared to CNT electrodes without fillers, while the stretchability of the composite electrodes reached over 100%.

In addition to 1D CNT materials, 2D graphene exhibits extraordinary mechanical and electrical properties and ultralow thickness,^[^ ^77^, ^78^ ^]^ therefore, its applications as on‐skin electrodes have been widely explored.^[^ ^79^, ^80^, ^81^ ^]^ Typically, high‐quality graphene is obtained by chemical vapor deposition (CVD). Ultrathin graphene electronic tattoo electrodes (Figure 3d) fabricated through a “wet transfer, dry patterning” manufacturing process was demonstrated by Ameri et al.^[^ ^82^ ^]^ The large‐area graphene was continually grown on copper foil through the CVD method. The “Wet transfer” method referred to copper etching procedure after CVD to retain the high quality of graphene. And the “Dry patterning” referred to the process to implement filamentary serpentine shape electrode patterning on graphene by using a programmable mechanical cutter plotter. Those processes resulted in tattoo electrodes with a total thickness of 463 ± 30 nm, achieving conformal contact to the skin without any fracture or delamination for several hours. Furthermore, the electrode–skin interface impedance of the patch was comparable to that of commercial Ag/AgCl gel electrodes.

The reduction of graphene oxide (GO) is another effective approach to obtain graphene.^[^ ^83^ ^]^ Yapici et al. developed a simple three‐step dip–dry‐reduce method to fabricate graphene electrodes on textiles (Figure 3e).^[^ ^84^ ^]^ Nylon fabric was firstly dipped in GO solution, followed by thermal treatment to form GO cladding on the textile surface. Subsequent reduction of GO cladding was achieved by chemical treatment with hydrogen iodide or hydrazine. The fabrication steps were simple and efficient, and the process could be easily applied for the production of large‐area textiles through integration with roll‐to‐roll manufacturing. The studies showed that the correlation of the signals between the graphene‐clad textile electrodes and Ag/AgCl electrodes reached 97%. In addition, the conductivity of nylon after coating with the rGO layer increased to 4.5 S cm^−1^, significantly higher than previous conductivity (6 × 10^−12^ S cm^−1^). However, the chemical reduction agents of GO, such as hydrogen iodide or hydrazine, are highly poisonous, facile, and environmental‐friendly fabrication processes are still needed. To tackle this challenge, our group developed a solution‐based approach to simultaneously reduce and pattern GO to fabricate graphene EP electrodes.^[^ ^85^ ^]^ Copper thin film sputtered on silicon substrate was utilized to reduce GO into rGO due to their lower redox potential than that of GO. The adhesive PDMS provided sufficient adhesion for rGO electrodes to achieve conformal contact with skin. It was shown that the rGO electrodes could capture EP signals accurately, including EOG, EEG, and EMG. Moreover, multichannel EMG electrode arrays as practical HMI devices were also presented through human hand gesture classification and control of a dexterous hand. It was pointed out that this approach was easily scalable for large area multielectrode fabrication without toxic chemicals, high temperature process, or photolithography patterning.

Besides the use of rGO as electrode materials, Sun et al. developed porous graphene to fabricate stretchable electrodes, as shown in Figure 3f.^[^ ^86^ ^]^ First, serpentine conductive trace of graphene was fabricated on PI films by using a CO_2_ laser‐patterning process. Porous graphene structures were formed synchronously due to laser heating. Subsequently, the laser‐scribed patterns were transferred on porous substrate to yield the electrode. The porous sponge substrate was manufactured by mixing silicone elastomer with sugar powders. It was demonstrated that the device had a low sheet resistance of 10.96 Ω sq^−1^ and a high failure strain of ≈330%. Due to the ultralow modulus (≈15 kPa) of the sample, the resistance of the device increased marginally (≈7%) at a bending radius of 2.5 mm for 500 cycles. Moreover, to further enhance the stretchability of the sample, kirigami cutting patterns were applied to the sugar‐templated sponge substrate. It was found that the resistance of the device only increased ≈1% after stretching up to 1000 cycles at 500% tensile strain. And the electrode was attached on skin for 7 days without inflammation, confirming its biocompatibility.

### 3.3 Electrodes Based on Metallic Nanomaterials

**Figure 4:**
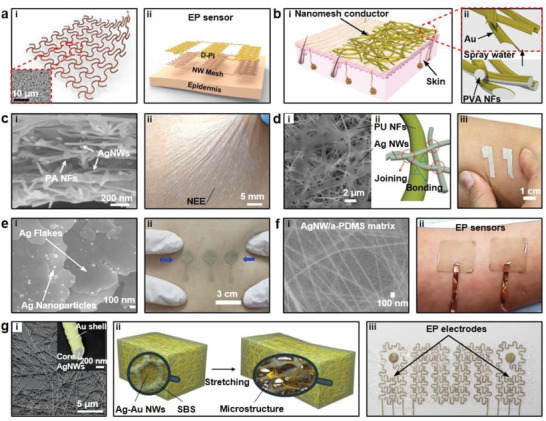
Electrodes based on Metallic nanomaterials. a‐i) On‐skin electrodes with Cu networked mesh structure (inset) and ii) schematic of Cu nanomesh electrodes. Reproduced with permission.^[^ ^98^ ^]^ Copyright 2016, Wiley‐VCH. b‐i) Au nanomesh conductors and ii) the process of the polyvinyl alcohol nanofibers (PVA NFs) dissolution. Reproduced with permission.^[^ ^44^ ^]^ Copyright 2017, Springer Nature . c‐i) A cross‐sectional SEM image of convoluted nanonetworks and ii) an image of the NEE intimately coupling with human skin. Reproduced with permission.^[^ ^100^ ^]^ Copyright 2019, Wiley‐VCH. d‐i) SEM image of Ag‐PU network conductors, ii) an illustration of the hydrogen bonding, and iii) porous Ag‐PU electrodes on skin. Reproduced with permission.^[^ ^101^ ^]^ Copyright 2019, Wiley‐VCH. e‐i) SEM image of the Ag flakes and Ag nanoparticle and ii) the ECCs‐based electrodes conformally attached to skin during compression. Reproduced with permission.^[^ ^102^ ^]^ Copyright 2019, American Chemical Society. f‐i) SEM image of AgNW‐embedded aPDMS matrix and ii) the AgNWs‐aPDMS ECG electrodes on skin. Reproduced with permission.^[^ ^103^ ^]^ Copyright 2018, American Chemical Society. g‐i) SEM image and backscattered electron (BSE) image (inset) of Ag–Au nanowires, ii) schematic illustrations of Ag–Au nanocomposites, and iii) photograph of Ag–Au nanocomposite EP electrodes. Reproduced with permission.^[^ ^104^ ^]^ Copyright 2018, Springer Nature.

A large number of researchers have demonstrated that metallic nanomaterials, such as nanowire,^[^ ^87^, ^88^, ^89^ ^]^ nanomesh,^[^ ^90^, ^91^, ^92^ ^]^ nanoparticle,^[^ ^93^ ^]^ and nanobelt,^[^ ^94^ ^]^ have high electrical conductivity. Therefore, metallic nanomaterials have great promise to function as building blocks for on‐skin electrodes.^[^ ^95^ ^]^ However, previous research reported that some metallic nanomaterials could be extremely brittle with ≈1% strain at break.^[^ ^96^ ^]^ Moreover, the impedance of metallic nanomaterials increased when subject to the tensile strain. Hence, it is of importance to enhance the conductivity and flexibility of the metallic nanomaterials simultaneously. One common method is to deposit metallic nanomesh onto elastic polymer substrate to enhance flexibility. As an example, Seo et al. prepared flexible Au nanomesh microelectrodes on flexible Kapton substrate.^[^ ^97^ ^]^ The Au nanomesh was fabricated by adopting the nanosphere lithography approach. Subsequently, the nanomesh was deposited on Kapton substrate by using the air/water interface through a self‐assembly method. And photoresist SU‐8 was spin‐coated on the top surface of Au nanomesh to form the microelectrode. The result indicated that after 300 bending cycles with a bending radius of 4 mm, there was no change in impedance of the Au nanomesh electrode. In addition, the normalized impedance of the Au nanomesh electrode was 8.14 Ω cm^2^, which was 2–10 times lower than that of the ITO or graphene electrodes. Han et al. reported ultrathin metal nanomesh decorated with highly flexible diluted polyimide (D‐PI) substrate.^[^ ^98^ ^]^ The metal nanomesh was patterned into serpentine structure through photolithography and etching methods to increase flexibility, as shown in **Figure** **4**a. Both CuNWs or AgNWs were studied due to their high electrical conductivity and excellent elongation. The studies presented that the nanomesh‐based elastomer had an excellent stretchability of ≈80% and an ultrathin thickness of ≈1.2 µm. Meanwhile, they almost maintained the absolute resistances during stretching and releasing cycles under 80% tensile strain. When the CuNWs‐based elastomer was adopted to prepare on‐skin electrodes (Figure 4a), the CuNWs electrode recorded high‐quality EMG and ECG signals, on par with Cu thin film‐based electrodes.

Electrospinning has been widely adopted to fabricate metallic nanomaterials‐based stretchable electrodes as it provides a versatile process to create functional nanofiber layer to support the conductive nanomaterials. Miyamoto et al. made a substrate‐free electrode with high conductivity and stretchability by synthesizing the ultrathin Au/PVA nanomesh layer (Figure 4b).^[^ ^44^ ^]^ The nanomesh layer was manufactured in two steps. In the first step, a PVA solution was electrospun and intertwined to form a mesh‐like NFs, followed by deposition of a 70–100 nm thick Au layer. Subsequently, when the nanomesh conductors were attached to the skin and sprayed with water, the PVA NFs dissolved and the nanomesh conductor attached to skin to function as electrodes (Figure 4b). It was pointed out that the dissolved PVA nanofibers formed a super‐thin adhesion layer with a thickness of several tens of nanometers, leading to highly conformal contact. And the nanomesh conductor had a low resistivity of ≈5.3 × 10^−7^ Ω m and a stretchability of over 40%. As expected, the contact impedance of the ultrathin Au nanomesh EMG electrode was comparable to that of standard gel electrodes. Besides, the amplitude of EMG signals measured by the Au nanomesh electrode was ∼1 mV, close to those measured by the gel electrode. During long‐term monitoring tests, there was no negative effect on the skin attached to the nanomesh electrode for 7 days, confirming the biocompatibility of the electrode. However, the conductance of the nanomesh changed sharply upon elongation and cycling stretch tests led to significant increase of resistivity, indicating relatively low robustness and durability. Fan et al. reported a highly robust and durable, ultrathin electrode composed of AgNWs and electrospun polyamide (PA) substrate.^[^ ^99^ ^]^ The electrode scaffold was formed by electrospinning PA6 NFs to improve the robustness of the nanonetwork. And the AgNWs were embedded into the PA6 scaffold by the vacuum filtration approach. The nanocomposite exhibited an extremely low sheet resistance of 8.2 Ω sq^−1^ and excellent optical transmittance of 84.9% at 550 nm. In addition, due to the reinforcement from nanofiber scaffold, the electrode still maintained robustness when distorted cyclically and soaked in saline solution. Specifically, the resistance of the device increased by less than 0.1% at the maximum bending of 300 m^−1^ for 3000 cycles. Owing to the ultralow thickness (300 nm) of the electrode, strong van der Waals force ensured that the electrode was in conformal contact to skin. The same research group also used AgNWs and PA NFs to fabricate a mutually convoluted nanonetwork epidermal electrode (NEE) (Figure 4c).^[^ ^100^ ^]^ PA NFs were electrospun, while AgNWs as conductive materials were simultaneously electrosprayed onto the same collector to form the electrode. It was noted that AgNWs were infiltrated across the nanofiber scaffold instead of distributed only on the top surface. The NEE showed a low sheet resistance (≈4 Ω sq^−1^) and excellent optical transmittance (≈82%). Owing to the nanonetwork as a robust scaffold that withstood stress caused by deformation, the NEE had a stable electric resistance which varied within 1.2% even after 50 000 bending cycles. Due to their ultralow thickness (125 nm), the epidermal electrodes were conformally integrated with skin via van der Waals force (Figure 4c). Surprisingly, the contact impedance of NEE was over 50% lower than that of commercial gel electrodes. Compared with ECG signals measured by commercial gel electrodes, the signal collected by the NEE was much more stable even during body motion. Another approach to enhance the stability and durability of the stretchable electrode is to create strong bonding between metal nanowires and electrospun NFs. Jiang et al. developed a porous nanomesh‐type elastic composite with a layer‐by‐layer structure to yield on‐skin electrodes with high durability, as illustrated by Figure 4d.^[^ ^101^ ^]^ The electrode materials consisted of AgNWs adhering to stretchable polyurethane (PU) NFs via interfacial hydrogen bonding with thin poly(vinylpyrrolidone) (PVP) layer on the surface of AgNWs (Figure 4d). The result indicated that the porous nanocomposite had excellent conductivity (9190 S cm^−1^) and stretchability (310%), exhibiting 82% resistance increase after stretching up to 1000 cycles at large tensile strain of 70%. The SNR of ECG and EMG signals (24.31 and 28.30 dB) obtained by the porous electrode was slightly lower than that of AgCl gel electrodes (24.86 and 28.92 dB, respectively) due to smaller effective contact areas as a result of its nanostructures. A bandage‐type EP sensor with integrated wireless communication modules on textile was demonstrated by adding textile substrate to the bottom of the electrodes by hot‐press process. And the conductivity and stretchability of the textile electrodes had no change under tensile strain within 200%. Moreover, it was found that the textile electrodes maintained low resistance (0.6 Ω) after water infiltration for 4h, exhibiting great potential to function as washable electrodes.

Stretchable electrode materials can be synthesized as conductive composites through the mixture of metallic materials with elastic polymers to achieve the balance of flexibility and conductivity. Our group developed an electrically conductive composites (ECC) to fabricate high performance on‐skin electrodes.^[^ ^102^ ^]^ The ECCs were synthesized in three steps. The surfactants on the commercial silver flakes were iodized using Potassium iodide (KI). Then, these silver iodide nanoparticles were converted to silver nanoparticles by photo exposure, as shown in Figure 4e. Afterward, stretchable ECCs can be synthesized simply by mixing the treated silver flakes with a polymer matrix. The studies showed that the PDMS–ECCs electrode had a resistivity of 9.43 × 10^−5^ Ω cm when filled with 75 wt% of treated silver flakes, while the resistivity was 7.04 × 10^5^ Ω cm if pristine silver flakes without the above iodization were used. The composites could be used to facilely fabricate electrode by adopting the screen‐printing method on the PDMS or ecoflex substrate. The composite electrodes achieved robust contact with the skin during deformable conditions (Figure 4e). And the SNR of Ecoflex‐ECC electrodes was ≈10.57 dB when recording ECG signals, comparable with that of the commercial Ag/AgCl electrodes (11.05 dB). It was noted that the matrix‐independent KI treatment of silver flakes provided a generic approach for the fabrication of a variety of highly conductive electrodes using various polymer matrices. Kim et al. also prepared AgNW‐embedded aPDMS matrix to fabricate ECG electrodes, as presented in Figure 4f.^[^ ^103^ ^]^ Among them, the composite was synthesized through mixing AgNWs with PDMS and a nonionic surfactant, Triton X. The polar functional groups present in Triton X interacted with the Pt catalyst present in the PDMS curing agent, thereby hindering the crosslinking reaction of PDMS, leading to aPDMS. Meanwhile, AgNWs were also effectively dispersed in the aPDMS matrix due to strong interactions between the polar functional groups of Triton X and AgNWs. It was found that the patch had low modulus (≈40 kPa) and high conductivity (35 Ω sq^−1^). Furthermore, the failure strain of the composite was enhanced to over 400%. Owing to enhanced stretchability and conformal contact with skin, the ECG sensors fabricated using the composite exhibited significantly improved performances compared with those of bare PDMS‐based counterparts. Choi et al. developed highly conductive, stretchable nanocomposite electrodes through the dispersion of Ag–Au nanowires in styrene–butadiene–styrene (SBS) in Figure 4g.^[^ ^104^ ^]^ The Ag–Au nanowires were manufactured by depositing the Ag nanowire surface with thick gold sheath. Hexylamine‐decorated Ag–Au nanowires, the SBS solution and additional hexylamine in toluene were combined and cast on a glass mold under ambient conditions, followed by solvent evaporation to obtain the microstructured Ag–Au nanocomposites composed of Ag–Au nanowire‐rich regions and SBS‐rich regions. It was pointed out that the cushioned microstructures ensured softness and stretchability during large deformation. As a result, the nanocomposites exhibited an optimized conductivity of 41 850 S cm^−1^ (maximum of 72 600 S cm^−1^) and stretchability of 266% (maximum of 840%) due to the high aspect ratio and percolation network of the Ag–Au nanowires. Owing to the Ag–Au nanowire core‐sheath structure, the silver ion was prevented from leaching to achieve biocompatibility. After the composites were implanted into the cardiac muscle for three weeks, the fibrotic reaction and inflammatory of the Ag–Au nanocomposite was far lower than that of the Ag nanocomposite. Thanks to the high softness and conductivity, the nanocomposite electrodes exhibited lower interfacial impedance than Ag/AgCl gel electrodes, resulting in high SNR for on‐skin EP signal monitoring.

Flexible or stretchable structures were also proved to be effective in promoting the flexibility of on‐skin electrodes. Qi et al. designed an out‐of‐plane tripod PDMS structure to achieve suspended Au nanobelts as highly stretchable electrodes.^[^ ^105^ ^]^ The stretchable electrodes were realized by transferring planar gold nanobelts onto the tripod PDMS substrate, and smooth sinusoidal structure was generated on Au nanobelts when the prestretched tripod PDMS substrate was relaxed. The tripod PDMS provided sufficient space for the Au nanobelts to deform upon tensile loading, leading to significantly enhanced stretchability (130%) of the electrodes. Moreover, there was no obvious increase in the resistance of electrodes after more than 10 000 stretch/release cycles. Won et al adopted the ancient art concept kirigami to fabricate transparent electrodes with tunable stretchability.^[^ ^106^ ^]^ The prepared AgNWs and colorless‐polyimide (cPI) layers on the glass substrate were patterned efficiently by using the laser ablation method to form the kirigami structure. By varying the cutting parameters on the CAD program, the stretchability of kirigami structure could be tuned to accommodate skin‐modulus of different body locations, human size, or applications. With this tunable feature, it was shown that the kirigami electrode could maintain strain‐invariant electrical properties when the loading tensile strain was ranged from 0% to over 400%. Meanwhile, the resistance of the electrode changed within 3% even after 10 000 stretching cycles, demonstrating excellent strain reversibility. The soft, thin and conformal features of the electrodes enabled the capture of various EP signals on curvilinear and irregular surfaces, with SNR 23% higher than that of conventional electrodes. The biocompatibility and stability of the kirigami electrode could be improved by coating the surface of AgNWs with Au to accommodate long‐term monitoring. Khan et al proposed freestanding structures of Au nanoparticle electrode arrays for increased flexibility to maintain conformal contact to skin.^[^ ^107^ ^]^ The electrode arrays were composed of the Au nanoparticles (AuNPs) printed on PEN substrate by inkjet‐printing process. Interestingly, freestanding flaps were cut around the electrodes using CO_2_ laser to enhance compliance, as these flaps were mechanically constrained from only one side. The inkjet‐printed AuNPs electrode exhibited minimum feature size of 62 *μ*m and a high conductivity of 5 × 10^6^ S m^−1^. Importantly, due to the design of the freestanding flaps, when the sensing depths increased from 1mm to >2 mm on skin, the percentage of successful contacts measured by the cutout electrode array remained >90%, which was significantly higher than that of flat electrodes without the freestanding flaps.

### 3.4 Other Materials for On‐Skin Electrodes

**Figure 5:**
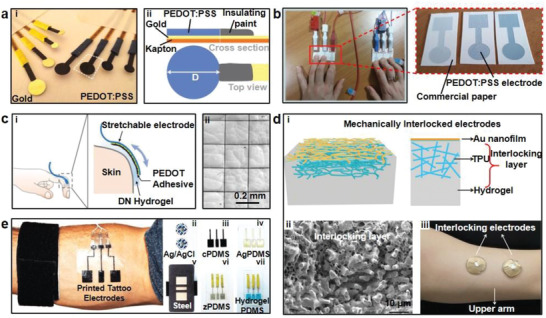
Other Materials for on‐skin Electrodes. a‐i) PEDOT:PSS electrodes with different diameters for EEG recording and ii) schematic illustrations show the cross section and top view of the electrode. Reproduced with permission.^[^ ^111^ ^]^ Copyright 2014, Wiley‐VCH. b) Printed PEDOT:PSS electrodes on commercial paper for ECG measurements. Reproduced with permission.^[^ ^112^ ^]^ Copyright 2017, Wiley‐VCH. c‐i) Schematics of the hybrid Au/hydrogel composite electrode on a forefinger and ii) SEM image of the Au arrays of 0.2 mm × 0.2 mm square tiles. Reproduced with permission.^[^ ^114^ ^]^ Copyright 2016, Elsevier. d‐i) Schematics of interlocked electrodes, ii) SEM of the interlocking layer, and iii) an image of interlocking electrodes on the upper arm. Reproduced with permission.^[^ ^115^ ^]^ Copyright 2020, Wiley‐VCH. e) A photo showing seven different types of electrodes including hydrogel‐PDMS electrode (Ag/AgCl and steel electrode as control groups). Reproduced with permission.^[^ ^116^ ^]^ Copyright 2019, Wiley‐VCH.

In addition to the materials mentioned above, other materials, such as conductive polymers,^[^ ^108^ ^]^ hydrogel,^[^ ^109^ ^]^ etc., have also been adopted for the fabrication of on‐skin electrodes. Owing to the biocompatibility, ease of deposition and relatively high conductivity of poly(3,4‐ethylenedioxythiophene) polystyrene sulfonate,^[^ ^110^ ^]^ Leleux et al. proposed a composite electrode composed of PEDOT:PSS and Au layers with different diameter from 0.5 to 1 cm (**Figure** **5**a).^[^ ^111^ ^]^ During preparation, the Au film was evaporated on Kapton substrates. And PEDOT:PSS film was deposited onto the Au layer through drop‐casting process to form the electrode. The result showed that the SNR of EEG signals obtained by the PEDOT:PSS electrode with a diameter of 8 mm was 24.4 dB, which was higher than that of the Au electrode (21.3 dB) and on par with the gel‐assisted electrode (24.9 dB). Bihar et al. developed ecofriendly ECG electrodes consisting of PEDOT:PSS and commercial paper, as shown in Figure 5b.^[^ ^112^ ^]^ The PEDOT:PSS ink was printed on paper substrate by inkjet‐printing method. Then the PEDOT:PSS polymer was printed one, two and three layers on the substrate to generate electrodes of three different thicknesses (85, 170, and 280 nm, respectively). The studies indicated that when the PEDOT:PSS thickness rose, the impedance of the electrode decreased and the SNR of the electrode increased correspondingly. And the electrode composed of three‐layer PEDOT:PSS polymers had the lowest resistance (≈1.04 × 10^6^ Ω) and the highest SNR for capturing ECG signals (11.01 ± 0.41 dB). Moreover, the electrode maintained high signal quality even after three months and there was only marginal change of the electrode resistance after more than 2000 bending cycles, indicating excellent measurement accuracy and durability.

Due to close similarity to biological tissues, hydrogels have attracted growing interest in the field of bioelectronics.^[^ ^113^ ^]^ A hybrid Au/hydrogel composite was prepared by Nagamine et al. to fabricate EP sensors.^[^ ^114^ ^]^ As illustrated in Figure 5c, the electrode consisted of three layers, namely an Au conductive layer, an adhesive PEDOT layer, and a double‐network (DN) hydrogel layer, from top to bottom. And there were preformed cracks in the Au array layers to promote the stretchability of the electrode, which led to stable resistance even during repeating stretch of 20% strain. Due to the presence of the PEDOT adhesive layer, there was large interfacial capacitance (9.5 ± 0.3 mF cm^−2^) at the interface of the layered composite to prevent interference from external noise and reduce electric impedance. As a result, the interface impedance of the hydrogel electrode was 27 times lower than that of bare Au film. Pan et al. prepared a hydrogel–elastomer hybrid through mechanical interlocking to fabricate on‐skin electrodes (Figure 5d).^[^ ^115^ ^]^ The interlocking layer was achieved by infiltration of hydrogel precursor into Au nanofilm‐coated porous thermoplastic polyurethane (TPU) (Figure 5d), and the bonding strength of the hydrogel–elastomer reached 50.9 J m^−2^, which was 14.3 times that of physically attached structures. It showed that the hybrid had an ultralow Young's modulus of 11.5 kPa, and failure strain of the hybrids reached up to 840% when the web thickness of the hybrids was 137 µm. The hydrogel–elastomer hybrid electrodes maintained absolute resistances when subject to 35% tensile strain. And the interlocking electrodes had lower interfacial impedance with skin than the Au/TPU electrodes, leading to higher SNR (43.03 dB) during the capture of EMG signals than that of the TPU/Au electrodes (17.8 dB).

Lopes et al. conducted comprehensive analysis on skin‐interfacing electrodes fabricated by different materials, including conductive composites made of PDMS as substrate and conductive particles such as carbon (cPDMS), silver (AgPDMS), anisotropic *Z*‐axis conductors made with silver‐coated nickel particles (zPDMS), a conductive tough hydrogel, as well as tattoo‐like adhesive poly(vinyl alcohol)‐coated films with stretchable biphasic Ag‐EGaIn electrodes (Figure 5e).^[^ ^116^ ^]^ The measurement performances of those electrodes were evaluated and also compared to rigid electrodes such as commercial Ag/AgCl and stainless steel electrodes. The results revealed that there was a direct correlation between conformity of skin‐electrode interface and SNR value. Additionally, the tattoo and hydrogel electrodes had the lowest contact resistance among all seven types of electrodes. Though the Ag‐EGaIn tattoo electrodes exhibited the highest SNR value and excellent stretchability of over 100%, owing to the ultrathin and fragile carrier film, it was challenging to integrate the electrode with integrated circuit (IC) chips to fabricate a practical biomonitoring patch. In contrast, hydrogel‐PDMS electrode was a preferred candidate for wearable systems due to the ease of integration of ICs with PDMS. Therefore, a wearable patch (110 mm long, 60 mm wide, and overall thickness of 1.5 mm) was developed based on the hydrogel‐PDMS electrodes. This study confirmed that hydrogels can be highly effective as electrode materials.

## 4 Adhesion to Skin

There are generally three methods frequently adopted to achieve adhesion with skin, namely directly using a biocompatible adhesive, fabricating various microstructures on the electrode to form a “dry adhesive,” and turning the electrodes into pressure sensitive adhesives (PSAs).

**Figure 6:**
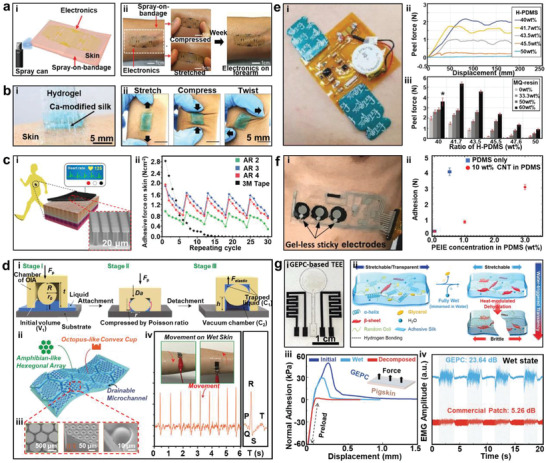
Electrodes with tuned adhesion. a‐i) Schematic illustrations of the liquid spray‐on‐bandage and ii) the multifunctional epidermal electronic system (EES) attached on forearm, the conditions of compression and stretch on skin and after wearing for a week. Reproduced with permission.^[^ ^118^ ^]^ Copyright 2013, Wiley‐VCH. b‐i) Schematic illustrations of Ca‐modified hydrogel patch, which was ii) firmly attached to the forearm when subjected to tensile, compression, and twisting forces. Reproduced with permission.^[^ ^121^ ^]^ Copyright 2018, Wiley‐VCH. c‐i) Illustrations of graphene‐based electrodes, ii) the SEM inset shows microstructures and comparison of adhesive force between 3M tape and bioinspired microstructures in repeated cycles. Reproduced with permission.^[^ ^76^ ^]^ Copyright 2017, American Chemical Society. d‐i) Adhesion model for OIA attachment, ii) schematic illustrations, and iii) SEM images of OIA structure, and iv) ECG signals recording on wet skin in dynamic movements. Reproduced with permission.^[ 135,136]^ Copyright 2017, Springer Nature; Copyright 2019, Wiley‐VCH. e‐i) Photograph of flexible ECG sensor on skin. Comparisons of peel forces of various silicone‐based adhesives with different ratios of H‐PDMS to V‐PDMS and MQ resin content. Reproduced with permission.^[^ ^139^ ^]^ Copyright 2017, Wiley‐VCH. f‐i) Photograph of the on‐skin device and ii) correlation between PEIE concentration and adhesion. Reproduced with permission.^[^ ^142^ ^]^ Copyright 2017, Wiley‐VCH. g‐i) Photograph of the GEPC‐based TEE and ii) schematic illustrations of the water‐triggered decomposition process, iii) comparisons of adhesion force before/during water treatment, and iv) the SNR of EMG signals obtained from the TEE and commercial patch during wet state. Reproduced with permission.^[^ ^150^ ^]^ Copyright 2019, Wiley‐VCH.

In the first method, a biocompatible adhesive is applied either between the electrode and the skin or on the electrode as an encapsulation. Both commercially available adhesives (e.g., water‐soluble tapes,^[^ ^117^ ^]^ silicone adhesives,^[^ ^56^ ^]^ medical liquid bandage,^[^ ^118^ ^]^ acrylic tapes^[^ ^119^ ^]^) and laboratory developed adhesives can be used. In the stretchable electrodes reported by Jang et al., ultralow modulus and adhesive silicone elastomer, Silbione RT Gel, was coated on an elastic fabric to form an compliant, breathable, and adhesive substrate.^[^ ^56^ ^]^ The degree of adhesion to skin (≈1.5 kPa) was higher than those of common silicones (Ecoflex, PDMS, and Solaris). Yeo et al. reported the adoption of spray‐on‐bandage as adhesive substrate for metal thin film electrodes, as illustrated in **Figure** **6**a.^[^ ^118^ ^]^ The liquid bandage could be easily sprayed onto thin film electrodes to form an ultrathin encapsulation (≈1 µm thick) of the device. It was demonstrated a 1.1 µm thick spray‐on‐bandage showed an average adhesion of 0.98–0.03 N, comparable with 3M medical tape (1.02 ± 0.01 N, ≈35 µm thick) of the same area (≈9 cm^2^). As a result, the integration with spray‐on‐bandage was also sufficiently robust such that they allowed repeated cycles of attachment and removal from skin, without damage to the electronics. Besides commercial adhesives or tapes, new formulations have been developed to provide additional functionality. Lee et al. prepared biocompatible gel electrodes by dispersing PVA in UV‐crosslinkable polyrotaxane.^[^ ^120^ ^]^ The device exhibited conformal contact and stable adhesion with a rat heart for more than 3 hours, which significantly improved the signal‐to‐noise ratio. The device could be removed after the PVA was dissolved. The adhesive strength, duration of contact, and conformability were controlled by changing the concentration of PVA. The experiment showed that with the increase of PVA concentration from 0 to 5 wt%, the peel force increased from 0.34 to 2.02 mN cm^−1^. Seo et al. proposed calcium (Ca)‐modified biocompatible silk adhesive to optimize the patch‐skin interface, as illustrated in Figure 6b.^[^ ^121^ ^]^ It was experimentally shown that silk fibroin had excellent adhesive characteristics due to the incorporation of Ca ions. The effective crosslink between Ca ions and random chains of silk via metal–chelate bonding and water‐capturing property of Ca ions enhanced the viscoelasticity and thus the mechanical interlocking of the silk film, leading to adhesive characteristics. The experiments demonstrated that the silk adhesive exhibited maximum peel strength of ≈800 N m^−1^ when the weight ratio of silk and Ca^2+^ was 70:30. It was shown that the metal electrodes coated with the Ca‐modified silk adhesive had a low impedance of 1.5 kΩ, comparable to that of metal electrodes decorated with commercial hydrogel (≈1.3 kΩ). Therefore, it was advantageous to use the silk adhesive for measuring ECG signals under motion due to strong peel strength and ionic conductivity. The ECG signals measured without silk adhesive exhibited large fluctuation and distorted signals during bending of the forearm. In contrast, the ECG signals recorded by the silk adhesive electrode maintained a stable state without motion artifacts even under cyclic bending conditions.

The second method is to create various microstructures by mimicking gecko feet,^[^ ^76^, ^122^, ^123^, ^124^ ^]^ octopus suckers,^[^ ^125^, ^126^, ^127^ ^]^ clingfish discs,^[^ ^128^ ^]^ mechanical interlocks^[^ ^129^, ^130^ ^]^ or other physical structures in the biological world to obtain the so‐called “dry adhesives.” The amazing adhesion of gecko foot to almost any surfaces is attributed to the large number of hierarchically arranged fibrils. According to the classic Johnson–Kendall–Roberts (JKR) theory, when an elastic element with spherical tip of radius *R* is pressed against a plane surface, its pull‐off force will be

(1) $$\begin{array}{c} F=\frac{3}{2}\;\pi RW \end{array}$$

where *W* is the work of adhesion.^[^ ^131^ ^]^ Now if this element is divided into *N* elements with spherical tips, each element has a characteristic size of $R/\sqrt{N}$ and the total force is $F\;=\;N\;\times \left(\frac{3}{2}\pi \frac{R}{\sqrt{N}}\right)W\;=\frac{3}{2}\;\pi RW\sqrt{N}$. Therefore, the adhesion strength increases by $\sqrt{N}\;$as the size of fibrils decreases, which is called contact splitting.^[^ ^132^ ^]^ Gecko‐inspired dry adhesive on soft substrate was demonstrated by Kwak et al. as early as in 2011.^[^ ^122^ ^]^ Mushroom‐like micropillars made of pure PDMS were fabricated but used as fixation unit rather than the electrodes of the skin patch. The mushroom shape is most effective among the variety of tip geometries tried so far. It removes the stress singularity at the contact edge and forces cavitation under the fibril so that to change the failure mode to one requiring higher force.^[^ ^132^ ^]^ Kim et al. directly prepared carbon‐based conductive nanocomposite electrode with gecko‐inspired microstructures to enhance the adhesion, as illustrated in Figure 6c.^[^ ^76^ ^]^ The aspect ratio (AR) of micropillars was optimized during the adhesion tests. Larger AR is expected to enhance the adhesion due to the higher elastic energy dissipated at pull‐off. But it is also limited by the mechanical properties and surface coverage.^[^ ^133^ ^]^ The studies showed that the adhesive composite electrode at AR = 3 demonstrated maximum adhesion force of ≈1.3 N cm^−2^, even on rough human skin and after the patch was used for over 30 cycles. This AR value is consistent with prior studies on pure PDMS adhesives, whose optimum AR is around 3.5.^[^ ^134^ ^]^ Owing to the microstructures, the electrode also exhibited self‐cleaning capability, as the micropillars with spatula tips greatly increased the contact angle between the surface and various liquid droplets. The stretchability and adhesive electrodes enabled long term and high‐ fidelity EP sensing while maintaining conformal contact with skin, even in underwater environment. The octopus uses muscle contraction to create a pressure difference between the inside and outside of the sucker, forming a closed vacuum to obtain adhesion to dry or wet substrates. A series of highly adhesive and water‐drainable skin patches with octopus‐inspired architectures (OIAs) were also developed by Pang et al., as presented in Figure 6d.^[^ ^125^, ^135^ ^]^ The adhesion of OIAs is composed of the suction stress and the van der Waals force, with the former playing the dominant role. When the OIAs gets pressed against a wet surface, the chamber is separated into the upper part and the lower part upon the contact between the dome‐like microstructure and the adjacent sidewalls. The capillary force drains the liquid toward upper part. After the removal of the external load, the elastic relaxation generates an extremely low pressure zone in the lower part. The suction stress *σ*_suc,wet_ is dependent on the material properties, geometric parameter and the preload force^[^ ^125^ ^]^

(2) $$\begin{array}{c} \sigma_{\mathrm{suc},\mathrm{wet}}≅-2.145\left(\sqrt[{3}]{\frac{1-v^{2}}{E}}\right)\left(\frac{\left(2R+l\right)Rkn}{\sqrt[{3}]{tr_{0}}}\right)\Delta P\times F_{P}^{1/3} \end{array}$$

where *ν* and *E* are the Poisson's ratio and Young's modulus of the material; *R* is the radius of OIA, *l* is the initial distance between the tip of the dome‐like structure and the substrate, *k* is the yield of OIAs, *n* is the number of the OIAs per unit area, *t* is the depth of the chamber and *r*_0_ is the radius of the dome‐like structure; *F*_p_ is the preload. They first fabricated an ECG electrode with the OIA structure using carbon black/P3HT/PDMS composite.^[^ ^127^ ^]^ In a later study, the OIA units were embedded on the top surface of the tree frog toe pad inspired hexagonal structures to further improve the adhesion.^[^ ^135^ ^]^ Due to the hexagonal microchannel network, the water residue was effectively discharged when the device was attached to sweaty skin, to guarantee strong adhesion. In comparison to the amphibian‐like patches with cylindrical holes, hexagonal microchannels, and a flat device, the amphibian‐like patches with OIA units demonstrated the strongest adhesion when attached to a Si wafer in all conditions (moist: 5.3 N cm^−2^; dry: 6.6 N cm^−2^; underwater: 4.5 N cm^−2^) even after 10 000 attachment–detachment cycles. And the bioinspired patch also showed the highest adhesion when mounted on sweaty or dry skin (sweaty: 1.92 N cm^−2^; dry: 2.36 N cm^−2^). When the hierarchically architectured patch was coated with rGO, it could still maintain high level of adhesion and was successfully used for detecting ECG signals even in wet skin surfaces during dynamic movements.

The third method is to chemically modify the substrate or the electrode to increase the tackiness, most often forming a PSA. Similar to dry adhesives, the adhesion of PSA does not depend on the chemical bonding, but is derived from the viscoelastic energy dissipation of the polymeric materials. Therefore, it is able to adhere to almost any surfaces including the skin or organ surface instantaneously upon the application of a light pressure at room temperature, which is particularly attractive for on‐skin electronics. Tse proposed that a PSA performance (*Г*) is composed of three components^[^ ^136^ ^]^

(3) $$\begin{array}{c} \Gamma \;=P_{0}\;BD \end{array}$$

where *P*_0_ is the intrinsic adhesion that is the thermodynamic work of adhesion, which is substrate dependent. *B* is the bonding term. Bonding usually occurs at a low frequency, if the storage modulus is below a certain level (in the Dahlquist criterion, this value is 3 × 10^5^ Pa), the PSA is less elastic and therefore has sufficient flow characteristics to wet the substrate surface. *D* is the debonding term and represents the energy dissipation process during the debonding process, which occurs at a much higher frequency than that of bonding. Chang et al. established viscoelastic windows (VW) to correlate the performance of PSAs with their viscoelastic properties.^[^ ^137^ ^]^ One general criterion for a good performance PSA is the low bonding plateau modulus at bonding (low) frequency and high‐energy dissipation at the debonding (high) frequency.^[^ ^138^ ^]^ PDMS is the most widely used substrate for flexible electrodes for its stretchability, softness, biocompatibility, breathability and stability. But the original storage modulus of fully cured PDMS is generally in the order of 10^6^ Pa.^[^ ^137^ ^]^ Various methods have been demonstrated to reduce the storage modulus to meet the Dahlquist conditions. Lee et al. prepared PDMS PSA by adding silanol‐terminated PDMS as the tackifier in the addition curing reaction between vinyl polydimethylsiloxane (V‐PDMS) and hydride‐terminated polydimethylsiloxane (H‐PDMS).^[^ ^139^ ^]^ By increasing the molecular weight of H‐PDMS and the content of tackifier, and reducing the ratio of H‐PDMS/V‐PDMS, the peel adhesive force on skin could reach more than 2 N, as illustrated in Figure 6e. Jeong et al. added a small amount of polyethyleneimine (PEIE) to PDMS as an inhibitor to reduce the crosslinking density of PDMS, and obtained a maximum adhesion of 1.2 N.^[^ ^140^ ^]^ Kim et al. used reversible micelles containing amino alcohol as an inhibitor to reduce the crosslinking density, and the obtained adhesive force can be adjusted from 0.014 to 1.1 N cm^−1^.^[^ ^141^ ^]^ The above two studies both reduced the degree of crosslinking of PDMS through the deactivation of Pt catalyst. In addition to the PSA substrate, electrodes based on PDMS conductive composites can also be made into a PSA with similar methods. Yamamoto et al. reported a gel‐less sticky CNT/PDMS electrode (Figure 6d) mixed with PEIE to reinforce adhesion, as illustrated in Figure 6f.^[^ ^142^ ^]^ The adhesion force of the electrode increased with the increase of the PEIE content. When 10 wt% CNT was added in PDMS, the maximum PEIE content allowed was around 3 wt%, leading to adhesion of 3 N cm^−2^ to skin. The adhesion achieved was quite stable as there was no decrease after 100 cycles of ECG measurements. Kim et al. promoted the adhesion of the AgNWs/PDMS electrode patch by adding nonionic surfactant (Triton X).^[^ ^103^ ^]^ Due to the interaction between the polar functional groups in Triton X and the Pt catalyst in the PDMS curing agent, the crosslinking reaction of PDMS was hindered, leading to high adhesion of the aPDMS. It was shown that the adhesion force of the composite electrode with 0.4% weight percent of Triton X reached ≈30 N m^−1^ at 40 °C.

Controllable adhesion to skin is essential for on‐skin electronics. The strong adhesion may cause pain or secondary injury when the electrodes were detached from skin after use.^[^ ^143^ ^]^ Hence, it is ideal that the interface adhesion can be tuned such that the device can be worn tightly and peeled off easily for multiple cycles. And many researchers have successfully demonstrated the control of adhesion by changing conditions, such as humidity,^[^ ^144^, ^145^ ^]^ temperature,^[^ ^146^ ^]^ magnetic direction,^[^ ^147^ ^]^ voltage,^[^ ^148^ ^]^ light,^[^ ^149^ ^]^ etc. Zhang et al. prepared a transient epidermal electronic (TEE) system embedded with the degradable protein‐based substrate to achieve tunable adhesion.^[^ ^150^ ^]^ As shown in Figure 6g, the framework of the substrate was based on a genetically engineered plasticized copolymer (GEPC), with silk fibroin as framework, resilin protein as the modifier and glycerol as the plasticizer. The combination of silk and resilin protein resulted in desirable properties such as high biocompatibility, flexibility, adhesion and low stiffness. When the substrate was rinsed with a large amount of water, the glycerol was replaced by the water molecules without reducing the electrical conductivity. After the extraction and drying process, the GEPC substrate became brittle after extreme shrinkage. When applied with minimal force, the GEPC substrate was fragmented to result in a decrease of adhesion. This study demonstrated a successful strategy for the development of on‐skin substrate for tunable adhesion. The adhesion after fully wet (29.19 kPa) was significantly lower than the original adhesion (49.36 kPa), and reached a nearly negligible state after water treatment (2.13 kPa). It is worth emphasizing that the partially wet state (e.g., sweat excretion) was not enough to trigger the decomposition process. Therefore, the TEE based on a GEPC substrate could still be used to record EMG signals under sweat‐infused states. Notably, the TEE exhibited a higher SNR of EMG signals (23.64 dB) than commercial EMG patches (5.26 dB), as shown in Figure 6f, due to the conformal contact. And after the water‐triggered decomposition, the peeling force of GEPC‐based patch dropped significantly.

## 5 Breathability

**Figure 7:**
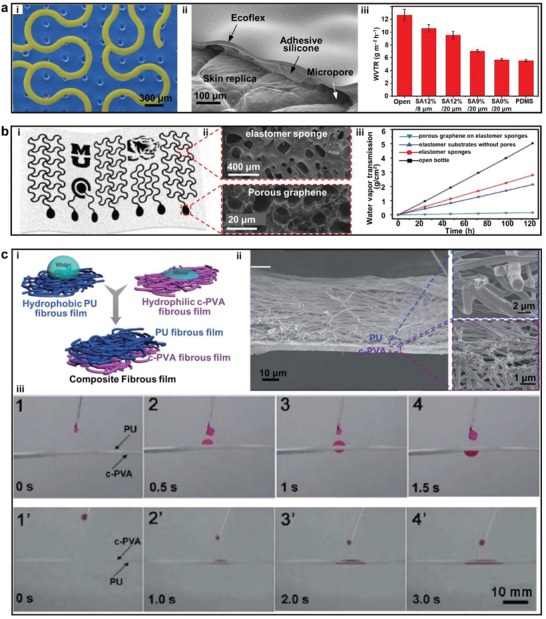
a‐i) SEM image of a microperforated silicone layer with patterned Au mesh electrode, ii) SEM tilted view of a bilayer microperforated silicone support laminated on skin replica, and iii) comparisons of WVTR values of different silicone films and the value without a film. Reproduced with permission.^[^ ^152^ ^]^ Copyright 2019, Springer Nature. b‐i) Photograph, ii) SEM image, and iii) WVTR of an EP sensor based on porous graphene and porous elastomer sponges. Reproduced with permission.^[^ ^86^ ^]^ Copyright 2018, Wiley‐VCH. c‐i) Schematic illustrations and ii) SEM image of dual‐layer PU/c‐PVA electrospun membrane, and iii) directional water transport from the hydrophobic (PU) side to the hydrophilic (PVA) side. Reproduced with permission.^[^ ^158^ ^]^ Copyright 2012, The Royal Society of Chemistry.

The residues of water vapor and sweat on the skin‐electrode interfaces likely lead to skin irritation and electrode failure during long‐term monitoring. Decorating soft electrodes with porous structures that offer breathable channels has been verified as a feasible path to address this issue. Among them, porosity which is related to pore size and pore density becomes one of the essential indicators for the breathability of porous materials. Besides, multilayer porous matrixes in contact with liquid results in capillary pressure. And the liquid will spontaneously osmose driven by capillary pressure difference. Affected by porous size and contact angle of the solid–liquid interface, capillary pressure of porous materials provides opportunities to further enhancing electrode breathability.^[^ ^151^ ^]^ In accordance with those mechanisms summarized in **Table** **1**, recent progress has gradually been made in developing breathable electrodes. For instance, Tian et al. fabricated a microperforated silicone layer as the support of Au mesh electrodes via microsphere‐template method, as shown in **Figure** **7**a.^[^ ^152^ ^]^ The micropores (diameter 80 µm) were obtained from the dissolution of a monolayer poly (methyl methacrylate) microspheres which were partially embedded in the near‐surface area of the silicone layer. Thus, the porosity of this silicone layer could be tuned by adjusting the density and size of these microspheres. Their tests also showed that increasing porosity of the microperforated silicone layer and decreasing thickness of the outer packaging layer could improve breathability of the support. In this work, the water vapor transmission rate (WVTR) of the most breathable support exceeded 10 g m^−2^h^−1^, which was two times higher than that of PDMS elastomer of the same thickness. Attributed to excellent breathability, the device was capable of recording stable, high‐fidelity EEG signals for more than five days of continuous wear, without any skin injury. Sun et al. not only prepared porous elastomer sponges as substrate by mixing silicone elastomer with sugar powders, but also introduced porous structures into sensing components (Figure 7b).^[^ ^86^ ^]^ Based on the mechanism that CO_2_ laser heating could convert polymer films into porous graphene, the bioelectronic sensors composed of graphene filaments were fabricated on PI films through a laser‐patterning process. During the process, only the regions of PI exposed to the laser were heated. Then, the patterned porous graphene was combined with elastomer sponges as bioelectronic sensor platform. A mass of interconnected channels provided by irregular pores expedited perspiration evaporation and gas/vapor diffusion. The WVTR of these platforms were up to 18 mg cm^−2^ h^−1^ at 35 °C, which was 18 times higher than that of silicone elastomers without pores. And no negative reaction happened when they were attached on the forearm skin of ten volunteers for 7 days, showing the long‐term biocompatibility.

**Table 1:** Characteristics of representative breathable electrodes

Materials/structures	Mechanism	WVTR [mg cm^−2^ h^−1^] ^a)^	SNR/contact impedance	Ref.
TPU porous film	Porous channel	23	ECG: 7.0 dB vs 7.1 dB (Conv.^b)^)	^[^ ^153^ ^]^
SEBS/multiscale pores	Porous channel	20.6	At 100 Hz: ≈33 kΩ vs ≈21 kΩ (Conv.)	^[^ ^13^ ^]^
Porous graphene/sponge	Porous channel/capillary force	18	At 100 Hz: 17kΩ vs 12 kΩ (Conv.)	^[^ ^86^ ^]^
Silicone/micropores	Porous channel	≈10	At 30 Hz: ≈30 kΩ	^[^ ^152^ ^]^
PU NFs	Porous channel	9.9	At 100 Hz: 160 kΩ vs 45 kΩ (Conv.)	^[^ ^101^ ^]^

^a)^ WVTR, Water vapor transmission rate;

^b)^ Conv., Conventional commercial Ag/AgCl electrodes.

Electrospinning was an efficient and low‐cost approach to construct porous nanofiber membranes with network structures. Furthermore, the fiber diameter, porosity and pore size of the electrospun membranes could be tuned easily by changing process parameters, such as the conductivity of solution, direct current voltage and fiber collecting distance. In recent years, utilizing electrospun fibers to develop ultrathin breathable electrodes with a porous structure similar to fabric/textiles has attracted increasing interest. For example, Fan et al. employed electrospun PA6 NFs (diameter 80 nm) as the scaffold of the electrode.^[^ ^99^ ^]^ The AgNWs (diameter 30 nm, length 30 µm) were embedded in PA6 scaffold through vacuum filtration, forming a conductive network. In addition, the thickness of this porous electrode was only 300 nm, which was difficult to realize by templating methods, reducing the distance for water vapor to diffuse into atmosphere. As a result, this electrode achieved a gas permeability of 6 cm^3^/s/cm^2^ at a pressure drop of 300 Pa, which was comparable to woven fabrics. Another ultrathin porous electrode made of Au nanomesh with sparsely overlapping spaghetti‐like structures was fabricated by Miyamoto et al.^[^ ^44^ ^]^ The Au nanomesh was patterned by depositing a 70–100 nm thick Au layer on top of electrospun PVA NFs (diameter 300–500 nm). In this work, the water vapor permeability was estimated by measuring the weight loss of water when the electrodes were attached to the opening of a water bottle at 25 °C and 30% humidity for one week. This electrode had the same rate in the weight loss of water as the bottle without a nanomesh electrode, demonstrating higher degree of gas permeability than 1.4 µm thick PET films and 10 µm thick silicone films as substrate. In addition, Xu et al. also proposed a phase separation‐controlled synthesis method to prepare multiscale porous electrode substrate.^[^ ^13^ ^]^ During this process, polystyrene‐*block*‐poly(ethylene‐ran‐butylene)‐*block*‐polystyrene (SEBS) and isopropyl alcohol (IPA) were mixed into chloroform to fabricate the precursor solution. Subsequently, the precursor was coated on an aluminum foil and dried in ambience. Due to the evaporation of volatile chloroform, the IPA separated from SEBS to form nano/microscale droplets. And the porous SEBS substrate with hierarchical pores was obtained after IPA droplets evaporated. It was notable that the SEBS substrate exhibited a remarkable WVTR (0.0206 g cm^−2^ h^−1^) and hydrophobicity (water contact angle, 125 ± 4°). Owing to its high hemispherical solar reflectance and IR thermal emittance, the substrate also achieved around 6 °C cooling effect under a solar intensity of 840 W m^−2^ by decreasing heat absorption and maintaining heat dissipation.

Although the aforementioned electrodes exhibited excellent breathability under normal condition, when the user sweats excessively under hot and humid conditions, or after exercises, the electrode would be immersed in perspiration because of their inability to quickly remove sweat and fail to function. To address this problem, functional textiles in sportswear for continuous sweat release might be used. These fabrics possess directional water transport property, and sweat could be transferred from skin to the outer surface of the fabric for quick evaporation.^[^ ^151^ ^]^ In this case, antigravity water transport was driven by the increasing capillary force in thin porous materials.^[^ ^154^, ^155^, ^156^ ^]^ Theoretically, the capillary force was generated by Laplace pressure *P*_L_, which was given by the Young−Laplace equation

(4) $$\begin{array}{c} P_{L}=\frac{4\gamma \cdot \cos\theta }{D_{\mathrm{pore}}} \end{array}$$

where *γ* is the surface tension of liquid water, *θ* is the advancing water contact angle (WCA of the inner surface of capillary), and *D*_pore_ is the pore diameter of capillary channels.^[^ ^157^ ^]^ Thus, the pore size and surface wettability play decisive roles in one‐way water transport capability. Considering the advantages of electrospun NFs including adjustable porous structure and easily tailored wettability, multilayered fibrous membranes of different pore size and surface wettability can be fabricated via electrospinning to achieve directional water transport. For instance, Wu et al. proposed a dual‐layer PU/PVA electrospun membrane with unidirectional water‐penetration function (Figure 7d).^[^ ^158^ ^]^ Particularly, the hydrophilic PVA fibrous membrane was chemically crosslinked with glutaraldehyde to overcome water dissolvability before PU fibrous membrane was electrospun onto it. According to wettability measurement, the PU layer showed a hydrophobic property with a WCA of 142.2° while the PVA layer retained hydrophilicity with a WCA of 22.1°. Besides the hydrophobic–hydrophilic wettability difference, the pore size of PU layer was larger than that of PVA layer, forming capillary force gradient along thickness direction. As a result, water could spontaneously penetrate across this fibrous membrane from the PU side to the PVA side, while its transport was blocked in the reverse direction. Although those strategies have not been leveraged for the fabrication of functional devices, they can be promising for the development of breathable on‐skin electrodes which can actively drive water flow in unidirection.

## 6 Integrated On‐Skin Electrode Systems and Applications

Although most on‐skin electrodes presented so far can measure only a single channel of EP data, multichannel electrodes are usually needed to obtain comprehensive EP information. Meanwhile, it is important to integrate on‐skin electrodes with other functional modules (e.g., data processing, power sources, wireless communication, etc.) to form standalone wearable systems for practical applications.^[^ ^159^, ^160^, ^161^, ^162^ ^]^ Therefore, we highlight the methods for the development of multichannel electrodes and integration strategies for standalone on‐skin electrode systems in this section. The applications of on‐skin electrodes in EP monitoring and HMI are also summarized in this section.^[^ ^163^, ^164^, ^165^ ^]^

### 6.1 On‐Skin Electrode System Integrations

#### 6.1.1 Large Area, Multichannel Electrodes

Although various on‐skin electrodes with high performance have been developed, these sensors usually recorded EP signals via a single channel (typically consisting of three electrodes including a measurement electrode, a reference electrode and a ground electrode) and the sizes of the electrodes were typically limited. As a result, the EP data from the single channel was rather limited as only muscle areas covered by the electrodes were measured. However, human EP signals are instable and complex due to the changing levels of crosstalk between adjacent muscles, inhomogeneities in muscle fiber activation, etc.^[^ ^166^ ^]^ Therefore, the single channel electrodes fail to capture EP characteristics from large areas of human skin or establish the correlation between spatial EP data and diverse human actions.^[^ ^167^ ^]^ Therefore, a large number of electrodes, usually in the form of electrode arrays for muscles of large areas, are typically adopted in the HMI systems to obtain comprehensive understanding of EP signals.^[^ ^168^ ^]^ In fact, some reports had verified that intensity and spatial distribution of high‐density EMG maps were very useful in improving the estimation accuracy of human intention and the strength of action.^[^ ^169^, ^170^, ^171^ ^]^ At the same time, multichannel electrodes could also alleviate the impact of electrode position shift and performance degradation caused by failure or error of certain electrodes in the array.^[^ ^172^ ^]^

**Figure 8:**
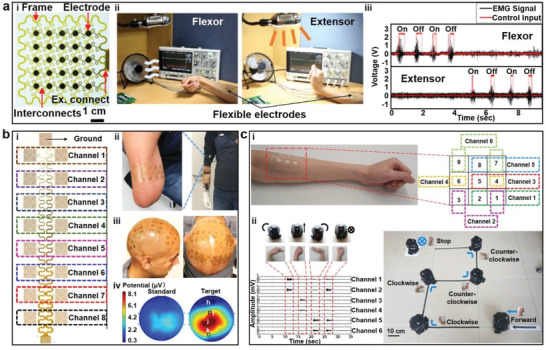
Large area, multichannel electrodes. a‐i) 5 × 5 interconnected electrode array ii) used for turning the fan or lamp on or off by squeezing the flexor or extensor muscles, and iii) two channel EMG signals measured from the flexor and extensor muscles (black) and corresponding control input signals (red). Reproduced with permission.^[^ ^59^ ^]^ Copyright 2016, American Chemical Society. b‐i) 8‐channel sensor array and ii) an array laminated on the amputated upper limb of a subject for controlling prosthetic movements, iii) four sensor arrays covering the scalp of the subject, and iv) the scalp ERP map. Reproduced with permission.^[^ ^152^ ^]^ Copyright 2019, Springer Nature. c‐i) 3 × 3 stretchable electrode array on the forearm and its 6 channels layout, ii) four gestures and the corresponding motion of the wheeled mobile robot. Reproduced with permission.^[^ ^102^ ^]^ Copyright 2019, American Chemical Society.

Kim et al. fabricated multichannel EMG sensor arrays by depositing and patterning metal layers (Ti/Au) with encapsulation of PI films.^[^ ^59^ ^]^ Mechanical support for the 5 × 5 interconnected electrode array (total size 71 mm × 68 mm) was provided by a 1.2 mm wide curvy frame (**Figure** **8**a). This design removed the backing layer covering the electrodes, reducing the restriction in deformation when stretched or compressed, leading to enhanced flexibility. Compared with the single channel system, multichannel electrode array could cover larger areas and acquire distinct signals from different muscles at the same time. In this study, two channel EMG signals captured from a flexor muscle and an extensor muscle on forearm were used to turn on and off a lamp and fan. Recently, Tian et al. reported ultralarge‐area electrode arrays (17 cm × 13 cm) formed by photolithography and etching of Cr/Au films.^[^ ^152^ ^]^ This high‐density sensor array consisted of 17 fractal mesh electrodes (1 cm × 1 cm), resulting in uniform coverage over all of the surface muscle groups in a residual limb. Due to the orderly arrangement of electrodes, the array provided eight channels of bipolar EMG signals with low crosstalk. These EMG signals allowed an amputee to control elbow, wrist and hand movements of his prosthesis, as shown in Figure 8b. Moreover, four sensor arrays (68 electrodes) could cover across human scalp completely to record EEG data with full‐scalp coverage. For the scalable fabrication of large‐area multichannel sensor arrays, printing processes such as screen printing have advantages in productivity and costs. Our group proposed a screen‐printing approach to fabricate a 3 × 3 stretchable electrode array with composite materials of silicone matrix and silver microflakes decorated with nanoparticles (Figure 8c).^[^ ^102^ ^]^ The relatively large area electrode array was highly stretchable and thin (total thickness ≈50 µm), maintaining conformal contact with skin even during stretch or compression with ≈10% strain. The multichannel electrode arrays also demonstrated great robustness and reliability as the array remained functional under 80% strain for over 1000 cycles. 6 bipolar EMG signal channels based on those 9 electrodes were adopted for signal capture and classification of the four hand gestures was easily achieved for remote control of a wheeled robot. In fact, four gestures can be identified by using only 2 channels of EMG data, the electrode array with 6 channels was capable of delivering more sophisticated control commands for HMI applications.

#### 6.1.2 Data Processing Unit Integration

**Figure 9:**
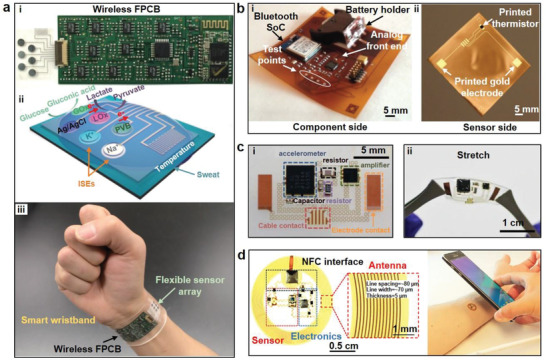
Standalone on‐skin systems through integration. a‐i) A flexible sensing array with integrated FPCB, ii) schematic of the sensor array for multiplexed perspiration analysis, and iii) the smart wristband worn on forearm. Reproduced with permission.^[^ ^174^ ^]^ Copyright 2016, Springer Nature. b‐i) The component side and ii) sensor side of an integrated wearable patch. Reproduced with permission.^[^ ^175^ ^]^ Copyright 2016, Wiley‐VCH. c) The mechanoacoustic–electrophysiological sensing i) platform and ii) its stretchability. Reproduced with permission.^[^ ^160^ ^]^ Copyright 2016, AAAS. d) The NFC‐enabled optoelectronic system. Reproduced with permission.^[^ ^181^ ^]^ Copyright 2016, AAAS.

The signals acquired by on‐skin electrodes need to be processed, temporarily stored and communicated with external devices for visualization or further analysis. Given that data processing cannot be achieved by utilizing soft material‐based devices due to relatively low computational performances, integration of conventional silicon‐based IC chips is a preferred solution to enable standalone on‐skin systems.^[^ ^173^ ^]^ However, due to the high modulus and hard nature of ICs, it is challenging to develop integration schemes to incorporate both soft materials and hard chips into one system and maintain functionality and reliability. In recent years, researchers had come up with effective strategies for the hard‐and‐soft hybrid system integration and those strategies can also be leveraged for on‐skin electrode system. For example, Gao et al. provided a flexible fully integrated wearable sensing system for in situ perspiration analysis, as shown in **Figure** **9**a.^[^ ^174^ ^]^ This noninvasive platform simultaneously monitored sweat metabolites (e.g., glucose and lactate), electrolytes (e.g., sodium and potassium ions) and the skin temperature. In this work, conventional silicon‐based ICs were mounted on a flexible printed circuit board (FPCB) for signal transduction, conditioning, processing and wireless communication. They chose an 8‐bit, low power microcontroller as the core of the system, which was compatible with the popular Arduino development environment and used to relay the signals to the Bluetooth transceiver. Therefore, by wearing this sensor system on forehead, wrists or arms, the user could read physiological data on an application of a mobile phone via Bluetooth connection. Similarly, Khan et al. reported a multisubstrate approach to fabricating flexible hybrid electronics for health monitoring (Figure 9b).^[^ ^175^ ^]^ Two gold ECG electrodes and a nickel oxide thermistor for measuring skin temperature were directly printed on flexible PI substrate, while silicon ICs and rigid passive components were mounted on the other side of the substrate. The two sides of copper circuit layers were fabricated by plated‐through holes to provide interconnection from the sensor side to the component side. It was noted that this approach, which minimized the size of sensor system and interconnect complexity, was compatible with the manufacturing process of FPCB industry. By further reducing the modulus of the substrates and using metal thin film as interconnection network, the systems obtained demonstrated not only flexibility but also stretchability.^[^ ^159^, ^176^, ^177^ ^]^ As an example, Liu et al. fabricated mechanoacoustic–electrophysiological sensing platforms for cardiovascular diagnostics and HMI (Figure 9c).^[^ ^176^ ^]^ Benefiting from the filamentary serpentine copper traces that interconnected chip components and encapsulation layer provided by silicone elastomer, the stretchability of this platform was up to 25%. By increasing the interconnect lengths or the thickness of the encapsulation layer, stress concentration in certain regions of the interconnects could be reduced to improve the stretchability of systems. Meanwhile, the low‐modulus characteristics of this device ensured conformal contact with skin, including curvilinear part of the neck.

A completely soft on‐skin electronic systems may also be possible through the fabrication of functional components, such as transistors, amplifiers, inverters and so on, with intrinsically soft materials, although their computational performances may be lower than ICs. For instance, Wang et al. fabricated inverters, Not‐AND (NAND) gates and amplifiers with intrinsically stretchable transistors composed of CNT electrodes.^[^ ^178^ ^]^ Son et al. developed stretchable resistive random access memory (RRAM) based on a sandwich structure of TiO_2_ nanomembrane/AuNP/TiO_2_ nanomembrane.^[^ ^179^ ^]^ At the system level, it should be noted that the power source is a challenging barrier to achieve standalone on‐skin electronics. Many wearable systems were powered by small rechargeable lithium‐ion batteries.^[^ ^174^, ^175^, ^180^ ^]^ However, due to the high‐power consumption of the circuit and wireless communication, battery life was typically no more than a few days. Besides the limited energy density, the battery usually appears as the largest hard component, impeding the miniaturization and flexibility of the system. To overcome the power challenge in wearable systems, near‐field communication (NFC) technology was investigated for simultaneous wireless data transmission and power delivery. For instance, Kim et al. reported battery‐free, wireless optoelectronic systems for multiwavelength optical characterization of the skin (Figure 9d).^[^ ^181^ ^]^ The circular loop antenna of this platform used 14 turns of copper lines with widths and thicknesses of ≈70 and 5 µm, respectively. An NFC reader (any NFC‐enabled smart phone, tablet, etc.) was able to acquire data at the sampling rate determined by the NFC chip employed. Another battery‐free monitoring system designed by Chung et al. showed high‐speed wireless data transmission based on the NFC antenna made of a magnetic loop.^[^ ^182^ ^]^ In this work, data stream rate was up to 800 bytes/s with dual channels and full‐coverage wireless operation also reached up to 25 cm in vertical direction.

### 6.2 Wearable Applications

#### 6.2.1 EP Monitoring for Healthcare

**Figure 10:**
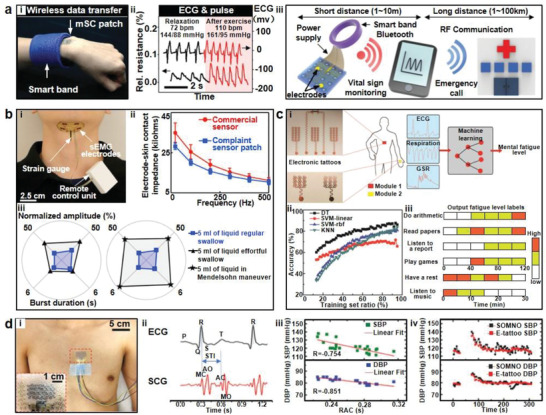
On‐skin electrodes for healthcare. a‐i) The mSC electrode system and ii) captured ECG and pulse signals, iii) the alarm function of the system. Reproduced with permission.^[^ ^183^ ^]^ Copyright 2016, Wiley‐VCH. b‐i) On‐skin sensor patch with remote control unit and ii) comparisons with commercial sensors, iii) pilot study on a patient diagnosed with Parkinson's disease and dysphagia. Reproduced with permission.^[^ ^189^ ^]^ Copyright 2019, AAAS. c‐i) On‐skin patches for recognizing mental fatigue, ii) prediction accuracy of different models, and iii) output fatigue level labels by the DT‐based model during daily mental works. Reproduced with permission.^[^ ^164^ ^]^ Copyright 2020, American Chemical Society. d‐i) The chest‐mounted E‐tattoo and ii) captured ECG, SCG and STI signals, iii) the correlation between SBP/DBP and RAC measured by an e‐tattoo, and iv) continuous estimation of SBP and DBP by the SOMNO and EMAC tattoo. Reproduced with permission.^[^ ^67^ ^]^ Copyright 2019, Wiley‐VCH.

A large number of studies have demonstrated that on‐skin electrodes become effective platforms for long‐term electrophysiological signal monitoring, such as ECG,^[^ ^75^ ^]^ EMG,^[^ ^86^ ^]^ EEG,^[^ ^66^ ^]^ and EOG.^[^ ^64^ ^]^ Among them, ECG signals are regarded as one of the most important heart health metrics. Therefore, a great deal of research effort has been devoted to collecting high‐quality ECG signals for personal healthcare. In addition to detecting high fidelity ECG signals, it is also important to provide the device with the capability of alarm function if abnormal ECG occurs in real time. In this regard, a wireless miniaturized suction cup (mSC) electrode system (**Figure** **10**a) was proposed by Choi et al. for monitoring and alerting cardiac arrhythmic diseases.^[^ ^183^ ^]^ Owing to the ultrathin thickness (<50 µm) and ultralow modulus (≈108 kPa), the patch was worn comfortably and recorded stable ECG and pulse signals without drift or distortion even in the exercise scenarios, as shown in Figure 10a. Due to the ultraconformal lamination of the patch, far‐field noises attenuated notably, ensuring that the QRS and T waves with low amplitude (≈2.5 mV) was clearly discriminated in ECG waveforms. Importantly, the on‐skin electrodes integrated in a smart band was wirelessly communicating with a smartphone via the Bluetooth unit. When the ECG signals were determined as abnormal states through the analysis software, an alarm signal was immediately triggered in the smartphone, transmitted the data to healthcare units simultaneously.

The EMG signals are widely present in the human body, and many diseases can be promptly diagnosed based on high‐precision EMG signals.^[^ ^184^, ^185^ ^]^ For instance, the abnormal EMG signals acquired from the chin are likely associated with head and neck cancer, resulting in swallowing difficulties.^[^ ^186^, ^187^ ^]^ In response to those symptoms, Constantinescu et al. developed ultrathin EMG electrodes for swallowing therapy.^[^ ^188^ ^]^ The flexible patch was attached to the chin and connected to the data acquisition system by using alligator clips. And the EMG signals was employed as biofeedback from muscle contractions during swallowing tasks. The results indicated that the on‐skin device was capable of accurately detecting swallowing events based on the features of the signal, for instance, the saliva swallows yielded short bursts of signal, while the Mendelsohn maneuver swallows led to prolonged muscle activity. And the quality of the signals measured by the patch was close to that of the Ag/AgCl electrode. It was found that the quality of the EMG signals obtained from the patch with an external reference electrode (8.467 ± 3.453 mV; 57.596 dB) was much higher than that of the patch with an integrated reference (0.433 ± 0.058 mV; 6.138 dB) in the saliva swallow task. Besides, the patch with an external reference also detected highly quality EMG signals (4.744 ± 4.547 mV; 31.213 dB) while no visible peaks were measured by the other one during head movements. It was proved that the on‐skin patch was particularly suitable for instructing patients on the correct swallow exercises. Considering the relatively low portability of wired patches and motion artifacts caused by the alligator clip used above, Kim et al. developed a wireless skin‐mountable sensor patch system (Figure 10b) for remote monitoring of dysphagia.^[^ ^189^ ^]^ The senor patch was composed of honeycomb‐ network electrodes and a strain gauge to detect EMG signals and strain waveforms. And the submental patch was connected to a potable self‐powered Bluetooth module by using a soft conductive film wire instead of alligator clips. During tests, the subjects were required to swallow liquid or budding in the effortful or regular way, compared with liquid using the Mendelsohn maneuver. It was found that electrode–skin contact impedances of the sensor patch were lower than that of commercial sEMG electrodes owing to intimate conformal contact (Figure 10b). In addition, the device collected high‐fidelity EMG signals, comparable to a commercial wireless unit (i.e., the correlation was over 0.95). Moreover, the corresponding EMG signal datum recorded by the patch were plotted as circular graphs. Among them, the upper hemispheres showed the value of the mean normalized amplitude while the lower hemispheres represented the value of burst duration. Through comparing the circular graphs, the submental muscle activity in different conditions were facilely distinguishable. As shown in Figure 10b, in contrast to regular swallows, the values recorded from effortful swallows were larger due to more vigorous chin muscle movements. Additionally, the signal via Mendelsohn maneuver exhibited 200% increase in amplitude and 270% increase in duration, in comparison to that via regular swallows. The result confirmed that the submental patch enabled the remotely accurate monitoring of the swallowing disorder. It was pointed out that the EMG data could provide clinically useful information to identify neuromuscular impairments, abnormalities in swallowing muscle activity and duration, as well as providing feedback during swallowing rehabilitation therapy.

Due to the fact that EOG signals are closely related to eye vergence motions, Mishra et al. developed a wireless skin‐like electrode system by combining a VR gear for mobile eye therapies.^[^ ^9^ ^]^ Among them, stretchable electrodes fabricated by an aerosol jet printing approach were mounted on the skin for collecting EOG signals. In conjunction with filters and classification algorithms, flexible circuit modules attached to the neck were utilized for processing raw EOG datum and identifying eye vergence. Besides, electrode positions and channels were further optimized to enhance measurement accuracy. During ocular vergence experiments, the wearable system integrated with a VR headset yielded higher classification accuracy (91%) than the physical apparatus (80%). To confirm the feasibility of home‐based vergence therapy, two ocular therapy programs (i.e., “Eccentric Circle,” “Brock String”) were conjugated onto the VR‐enabled wireless electronic system. The results demonstrated that three participants with no strabismus issues had difficulty in converging during initial tests (accuracy, ≈75%), which were successfully improved in later tests (final accuracy, ≈90%). Simultaneously, inconsistent EOG signals were precisely detected from a subject with strabismus exotropia during a divergence motion. Therefore, this periocular electronic system enabled the quantitative feedback of convergence insufficiency and strabismus.

Given that continuous focus of mental activities usually results in mental fatigue,^[^ ^190^ ^]^ EP signals are also capable of applications of fatigue analysis due to their noninvasive and convenience of real‐time analysis. Zhang et al. developed an artificial neural network (ANN) algorithms to efficiently analyze EP and electrobacterial‐graph (EBG) signals obtained from wearable devices.^[^ ^150^ ^]^ The 12 representative features of the ECG, EMG, EOG and EBG signals (3 features for each signal) were extracted and integrated as one input vector for the ANN algorithms. And the ANN was programmed by using 24 hidden layers and 4 output status (i.e., vigorous, exercising, tired, stimulated), corresponding to the status from active to fatigue. 160 vectors of data acquired from 10 subjects were utilized for training the ANN to enhance the classification accuracy. By comparing the output status of the algorithms with the actual status of human, the classification accuracy of the ANN algorithm was up to 96.9%. To validate this model, the wearable devices were applied in real‐life scenarios, such as running, and the changes of physiological states predicted in the model were almost identical to the subjective feeling of the participant. Additionally, the classified state also changed correspondingly after the subject drank caffeine as energy beverages. In contrast to the single ANN algorithm, Zeng et al. developed three types of machine‐learning algorithms to optimize the mental fatigue prediction model based on a multimodal on‐skin electrode platform (Figure 10c).^[^ ^164^ ^]^ The process consisted of three steps. First, three flexible electrodes and a strain sensor were mounted on the chest for detecting ECG signals and respiration rate respectively (module 1). And two flexible electrodes were attached to the palms for monitoring galvanic skin response (GSR) (module 2). When the subjects were required to finish requested mental tasks twice, 60 min of relative signals were collected in real time. Afterward, 6 kinds of different features were extracted from physiological signals. According to self‐report questionnaire scores, fatigue levels were divided into no fatigue, fatigue, and severe fatigue, which were combined with corresponding physiological features to set up the database. Finally, three machine learning algorithms including support vector machine (e.g., the SVM linear kernel and the SVM radial basis function kernel), K‐nearest neighbor (KNN) and decision tree (DT) were applied for model training and building fatigue prediction models based on the database. As shown in Figure 10c, the prediction accuracy increased by a higher training set ratio, wherein the fatigue prediction model established by the DT algorithm showed the highest accuracy of 89% at a training set ratio of 95%. Figure 10c showed that the DT‐based predictive model had successfully output corresponding fatigue levels when the subjects performed daily mental work for two hours, such as arithmetic, puzzle games, etc. As expected, the fatigue level predicted via the model was also alleviated after the subjects listened to music and closed eyes for a while.

EP signals can also be combined with other physiological signals to achieve more comprehensive diagnosis. For instance, cardiovascular health was not only associated with the ECG signal but also the mechanoacoustic signals, such as seismocardiogram (SCG),^[^ ^191^ ^]^ ballistocardiogram (BCG),^[^ ^192^ ^]^ and phonocardiogram (PCG).^[^ ^193^ ^]^ To provide more comprehensive insights into cardiovascular health, Ha et al. prepared a chest‐laminated electronic tattoo (E‐Tattoo) for monitoring ECG and SCG signals simultaneously, as shown in Figure 10d.^[^ ^67^ ^]^ The E‐Tattoo was composed of the Au electrodes and vibration sensors. Through the 3D digital image correlation (DIC) technique, the best position coupled with the patch was determined in the chest vibration map (i.e., the left edge of the middle of the sternum). As shown in Figure 10d, the prototype device recorded the clear peaks of the SCG and ECG waveforms synchronously during the signal test. And the systolic time interval (STI) was extracted from the measurement of SCG and ECG (i.e., R‐AC (aortic valve closing) intervals). It was worth noting that there was also a strong negative correlation between RAC measured by an e‐tattoo and systolic blood pressure (SBP)/ diastolic blood pressure (DBP) measured by SOMNO touch. To further validate the reliability of BP monitoring, combined with the corresponding calibration equation for each participant, their BP were calculated based on the RAC measured by the patch, which were almost consistent with the BP estimated by SOMNO during the Valsalva maneuver (Figure 10d). As a result, besides monitoring cardiovascular diseases, the E‐Tattoo also provided a novel path for diagnosing obstructive sleep apnea and coronary artery disease associated with BP. This example demonstrated that multimode physiological monitoring can enrich data collection and diagnosis through data fusion.

#### 6.2.2 Human–Machine Interfaces

EPs signals of the human body are directly correlated to the state of the human body, for example, EMG signals acquired from the arm vary by gestures, EOG signals are linked to eye movements, and EEG signals are correlated with mental status and external stimuli. As such, EP signals can be processed to quantitatively or qualitatively related to human intention, leading to applications in HMI. In fact, a large number of researchers have demonstrated that on‐skin electrodes are ideal EP signal platforms for applications in HMI.

**Figure 11:**
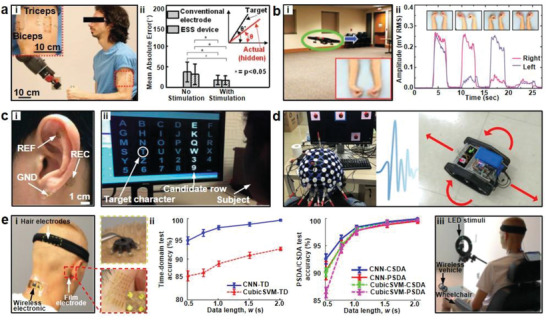
Applications of on‐skin electrodes in HMI. a‐i) On‐skin devices on the bicep and tricep (inset) during control of the virtual arm and ii) accuracy associated with a virtual arm targeting task. Reproduced with permission.^[^ ^55^ ^]^ Copyright 2016, Wiley‐VCH. b‐i) Quadrotor control by sEMG signals from forearms based on the control gestures and ii) sEMG signals acquired from forearms corresponding to four gestures. Reproduced with permission.^[^ ^61^ ^]^ Copyright 2013, Wiley‐VCH. c‐i) LTE electrodes for EEG recordings and ii) demonstration of the spelling task based on the BCI. Reproduced with permission.^[^ ^58^ ^]^ Copyright 2015, National Academy of Sciences USA. d) The AgPMS electrode BCI system for controlling a driverless car. Reproduced with permission.^[^ ^163^ ^]^ Copyright 2019, American Chemical Society. e‐i) Flexible wireless electrode systems, ii) CNN and SVM test accuracy on using time‐domain data and frequency‐domain data, and iii) in vivo demonstration of the on‐skin BMI with human subjects. Reproduced with permission.^[^ ^191^ ^]^ Copyright 2019, Springer Nature.

Since EMG signals can be easily acquired from muscle tissues, they are widely adopted for successful applications in HMI. For example, an on‐skin stimulation and sensing platform was designed by Xu et al. for sensorimotor prosthetic control (**Figure** **11**a).^[^ ^55^ ^]^ The systems were composed of multiple electrodes, used to collect EMG signals and offer neural stimulatory inputs. By attaching the patch to the subject's right arm, the researchers set the root‐mean‐square (RMS) value of EMG signals as the proportional control for the grip force value of the prosthesis. During the bottle gripping test, the subject successfully controlled the gripper at expected level of gentle touch with stimulation feedback, while the gripper likely resulted in the bottle to collapse without feedback. According to the type of electrode used (conventional electrodes or epidermal patches and whether there was electrical stimulation feedback), 4 groups of experiments were performed to evaluate the control error. By comparing the moving target angles of the virtual arm with the subject's actual angle of motion, Figure 11a shows that the mean absolute errors of angle were 37.2° and 31.6° respectively, when the stimulation feedback was not applied for the conventional and epidermal electrodes. While lower errors of 17.8° and 16.9° respectively, were observed with feedback. Therefore, when the feedback conditions were consistent, the epidermal electrodes showed lower angle errors than the conventional electrodes in sensorimotor prosthetic control.

Besides the application of prosthetic control, a wireless on‐skin sensor recording the EMG signals was used as a computer game controller by Kim et al.^[^ ^57^ ^]^ The wearable patch was attached to the throat to monitor muscle activity by collecting the EMG signals during speech. In the vocalization experiments, the on‐skin electrode measured four kinds of distinguishable EMG signals when the subject repeatedly vocalized four words (“up,” “down,” “left,” and “right”) for 10 times. Then the EMG datum were processed by dynamic time‐warping pattern‐recognition algorithms as the control commands of a computer game (Sokoban). The results showed that the time of signal recognition and classification was less than 3s with an accuracy of >90%. Jeong et al. also demonstrated the possibility of controlling a drone quadrotor (Figure 11b) based on the surface electromyography (sEMG) signals obtained from epidermal electrodes.^[^ ^61^ ^]^ They collected the corresponding sEMG signals when the subject showed four different bimanual gestures, as shown in Figure 11b. Then, the linear discriminant analysis (LDA) classification algorithm was applied to bin four sEMG signals into corresponding commands, such as In, Left, Right, and Out. It was found that the overall accuracy of classifications controlled with bimanual gestures was up to ≈91%, much higher than that with unimanual gestures (≈48%). Meanwhile, these commands were implemented to control the quadrotor for different motions (e.g., “take off,” “rotate,” “fly forward,” and “land”). Similarly, Won et al. reported kirigami electrodes utilized to control a quadcopter drone.^[^ ^106^ ^]^ The patches were mounted on the right and left forearms to record sEMG signals. The sEMG signals based on different gestures were transmitted to a computer via a WiFi network. Based on the custom classification algorithm, the RMS values of signals were classified as corresponding flight orders. The study demonstrated that the drone was successfully controlled to fly on desired routes by using the corresponding gestures.

Beyond EMG signals, Ameri et al. demonstrated the wirelessly control of a quadcopter via EOG signals collected by the graphene on‐skin electrodes.^[^ ^194^ ^]^ In this experiment, the subject was asked to wear four tattoo sensors when a gel electrode was laminated on the mastoid as a reference electrode. Two patches were mounted near outer corner of two eyes to record horizontal eye movement. The other two patches were attached above and below the left eye respectively to detect vertical eye movements. To acquire high‐precision EOG signals, the subject was required to move their eyes toward only one of light‐emitting diodes (LEDs) at five different directions at a given time, to guarantee at least 45° of eyeball movement. It was noted that the negative potentials were recorded by the patches when the eye looked downward or toward the right, while eyes moving to the left or upward resulted in positive potentials. Meanwhile, the amplitudes of the EOG signals recorded in the horizontal and vertical direction were 17.7 ± 1.5 and 12.8 ± 0.5 µV, respectively. With the signal datum wirelessly transmitted to a laptop, they were registered as control commands of the quadcopter. The accuracy of this HMI device reached 92 ± 2%. EOG signals acquired with soft‐fractal electrodes were also utilized for wireless wheelchair control by Mishra et al.^[^ ^195^ ^]^ Similar to the above method, the subject was required to wear vertical and horizontal electrodes on the corners of the eyes. Likewise, the real‐time EOG datum were wirelessly transmitted to a laptop interface via the Bluetooth unit. It was shown that when the eye moved up, down, left and right respectively, the corresponding EOG signals were converted to the motion instructions of a wheelchair including forward, stop, turn counter‐clockwise, and turn clockwise by using the classification algorithm. The studies proved that the accuracy of HMI with soft, fractal electrodes was 94.1%, higher than that with conventional rigid electrodes (91.9%).

EEG signals can be acquired with on‐skin electrodes in a noninvasive way, endowing them inherent advantages in the applications of BCI.^[^ ^196^ ^]^ Norton et al. designed EEG electrode systems to perform spelling tasks, as shown in Figure 11c.^[^ ^58^ ^]^ The demonstration devices included a text speller interface based on steady‐state visually evoked potentials (SSVEP) and event‐related potential (P300 wave) recordings,. Through a classifier based on canonical correlation analysis (CCA) algorithms, it was shown the on‐skin electrodes‐based approach could achieve a spelling rate of 2.37 characters per min, which was only a factor of two to three times slower than a full cap system that uses 8–10 electrodes on the hairy scalp (4–7 characters per min). SSVEP from a long‐term epidermal (LTE) electrode and that from a conventional electrode showed very similar patterns and amplitudes. In addition, the P300 event‐related potentials (ERP) recorded by the on‐skin device were easily distinguishable between target and nontarget stimuli. Lin et al. also proposed gel‐free nanowire/polyvinyl butyral (PVB)/melamine sponge (AgPMS) EEG electrodes (Figure 11d) for gaze/tracing/vision‐based selection and mind control of a driverless car based on the SSVEP.^[^ ^163^ ^]^ During the demonstration experiments, the hairless male participant was asked to wear the gel‐less electrode cap and conventional electrodes sequentially. The hairy female participant only wore the gel‐less electrode cap. They were asked to gaze at different flashing buttons at corresponding frequencies in the screen with SSVEP stimuli. The results showed that when the subjects stared at one of the target buttons, the highest peak amplitude of the EEG signals also appeared at the corresponding frequency. And the peaks of frequency spectrums recorded from the three participants were similar both in hairless and hairy conditions. Subsequently, by using the canonical correlation analysis (CCA), the correlation coefficient (CC) between analog signals and relevant EEG data was attained in the frequency domain. And the maximum CC value (CCmax) occurred in the target frequency was assessed as the correct discriminating result via the BCI algorithm. It was found that when slightly dropped to 84% on hairy skin, the BCI accuracy of the gel‐less electrode on hairless skin was 86%, comparable to that of conventional electrodes (88%). The male participant could finish the racing/vision‐based selection via the SSVEP‐based BCI. Based on the classifications of SSVEPs, EEG signals recorded at 6.00, 6.66, 7.50, and 10.00 Hz flickering stimuli corresponded to the commands of forward, backward, turning left, and turning right, and the female wearing the BCI cap successfully controlled the driverless car (Figure 11d). To further enhance the accuracy and portability of the BCI, Mahmood et al. developed a wireless on‐scalp electrode system for monitoring of the SSVEPs by combining the deep learning algorithm.^[^ ^197^ ^]^ The BCI system was composed of three parts, including flexible wireless data processing unit attached to the back of the neck, an ultrathin film electrode on the mastoid as the driven ground, and three flexible hair electrodes laminated on the scalp via a headband (Figure 11e). During the tests, when four LEDs evenly distributed on the ring flashed at unique frequencies, the subjects were asked to stare at four different LED locations and close eyes for alpha rhythms respectively. In the meantime, the flexible electrodes on the scalp recorded the EEG signals in real time. Subsequently, the deep learning algorithm enabled the classification of SSVEPs with only two channels of EEG datum. To enhance the accuracy of classification, the convolutional neural networks (CNNs) algorithm and CubicSVM algorithm were developed to perform the accuracy tests. Figure 11e showed that the optimized two‐layer CNN exhibited higher accuracy in all tests (i.e., time‐domain tests, power spectral density analysis (PSDA)/cross‐spectral density analysis (CSDA) tests). Finally, the classification outputs based on the CNNs algorithm were registered as the corresponding commands (alpha waves: stop; 11.1 Hz: forward; 15.2 Hz: rotate counterclockwise; 12.5 Hz: rotate clockwise; 16.7 Hz: reverse) to control a wireless wheelchair and a wireless mini‐vehicle. In addition, the BCI was also applied to control the presentation software with the corresponding commands (no change, start, next slide, previous slide, end). It was noted that three applications showed the high control accuracy of 94.01 ± 3.6% at 0.512s intervals and 96.24 ± 3.4% at 1.024s intervals, respectively.

## 7 Discussions and Perspectives

In this review, we have first highlighted desirable features of on‐skin electrodes. The latest advances in the development on‐skin electrodes are also summarized, with particular emphasis on electrode materials, including metal films, carbon‐based materials, metallic nanomaterials, and other materials such as hydrogel and conductive polymers. Afterward, recent research endeavors to improve the adhesion and breathability of on‐skin electrodes are also discussed as those properties are of great importance to enhance the measurement performances and wearable comfort of on‐skin electrodes. In addition, the integration schemes to incorporate electrode array and data processing units for standalone on‐skin electrode systems of high performances are also explored, followed by representative applications of on‐skin electrodes in the fields of personal healthcare and HMI.

**Figure 12:**
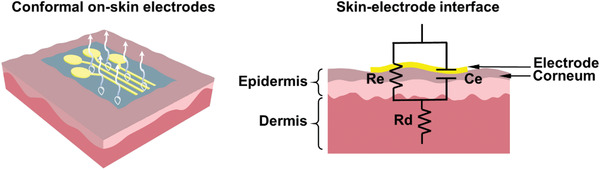
The skin‐electrode interface model of on‐skin electrodes.

The representative on‐skin electrodes reported so far are summarized in **Table** **2**. The electrode‐–kin interfaces are often depicted as a parallel circuit of leakage resistance and capacitance to evaluate the performances of EP electrodes, as shown in **Figure** **12**.^[^ ^198^ ^]^ The electrode–skin impedance can be derived as^[^ ^199^ ^]^

(5) $$\begin{array}{c} Z\left(\omega \right)=R_{d}+\frac{R_{e}/jC_{e}\omega }{R_{e}+1/jC_{e}\omega }=R_{d}+\frac{R_{e}}{1+j\omega C_{e}R_{e}},\;\omega =2\pi f \end{array}$$

**Table 2:** Summary of representative on‐skin electrodes

Materials	Conductivity/resistivity	Stretchability [%]	Modulus ^a)^	Thickness	SNR/amplitude/contact impedance	Ref.
Au /UL‐Sil/textile	/	≈220	≈3 kPa	≈605 µm	ECG: ≈0.4 mV; EMG: ≈5 mV	^[^ ^56^ ^]^
Au/PI/polyester	/	>30	≈60 kPa	≈37 µm	EMG: ≈100 µV vs ≈ 100 µV (Conv. ^b)^)	^[^ ^57^ ^]^
Au/PET/tegaderm	/	>55	≈7.4 MPa	≈60 µm	ECG: ≈0.8 mV vs ≈0.4mV (Conv.)	^[^ ^66^ ^]^
Au/plasticized silk	7 Ω sq^−1^	>400	0.1–2 MPa	≈160 µm	EMG: 17.17 dB vs 17.95 dB (Conv.)	^[^ ^60^ ^]^
CNT/aPDMS	10^−2^–10^−1^ S cm^−1^	>30	≈27.5 kPa	≈120 µm	ECG: ≈4 mV vs ≈4 mV (Conv.)	^[^ ^75^ ^]^
CNT/graphene	≈100 Ω cm	>100	2.3 MPa	≈15 µm	ECG (underwater): 0.2 mV vs 0 (Conv.)	^[^ ^76^ ^]^
Graphene/PMMA	/	>40	≈3.3 GPa	≈460 nm	ECG: 15.22 dB vs 11 dB (Conv.)	^[^ ^82^ ^]^
rGO/aPDMS	/	>20	≈27.5 kPa	≈50 µm	EMG: 16.8 dB	^[^ ^85^ ^]^
Graphene/silicone	10.96 Ω sq^−1^	>1000	≈15 kPa	≈500 µm	ECG: 24.1 dB vs 21.6 dB (Conv.)	^[^ ^86^ ^]^
AgNWs/SEBS	11000 S cm^−1^	>150	≈0.2 MPa	≈100 µm	EMG: ≈20.7 dB vs ≈22.4 dB (Conv.)	^[^ ^13^ ^]^
Au nanomesh/PVA	5.3 × 10^−7^ Ω m	≈48	/	≈70 nm	EMG: ≈1 mV vs ≈1 mV (Conv.)	^[^ ^44^ ^]^
AgNWs/PA NFs	≈4 Ω sq^−1^	>50	/	≈125 nm	At 10 Hz: ≈10^5^ Ω vs ≈3 × 10^5^ Ω (Conv.)	^[^ ^100^ ^]^
AgNWs/PU NF	9190 S cm^−1^	≈310	/	≈3 µm	ECG: 24.31dB vs 24.86 dB (Conv.)	^[^ ^101^ ^]^
AgNPs/ecoflex	9.43 × 10^−5^ Ω cm	>119	≈69 kPa	≈60 µm	ECG: 10.57 dB vs 11.05 dB (Conv.)	^[^ ^102^ ^]^
AgNWs/aPDMS	35 Ω sq^−1^	≈400	≈40 kPa	≈2 mm	ECG: ≈500 µV vs ≈450 µV (Conv.)	^[^ ^103^ ^]^
Ag‐AuNWs/SBS	72 600 S cm^−1^	≈840	≈3.28 MPa	≈20 µm	At 10 Hz: ≈10^3^ Ω vs ≈1.8 × 10^3^ Ω (Conv.)	^[^ ^104^ ^]^
AgNWs/GEPC	9.66 Ω sq^−1^	>50	<49.36 kPa	≈30 µm	EMG (wet): 23.64 vs 5.26 dB (Conv.)	^[^ ^150^ ^]^
PEDOT/hydrogel/Au	35 ± 5 Ω sq^−1^	>20	/	≈0.5 mm	At 10 Hz: ≈10^2^ Ω vs ≈10^3^ Ω (Au electrode)	^[^ ^114^ ^]^
TPU/hydrogel/Au	≈40.9 Ω sq^−1^	≈840	≈0.4 MPa	≈80 µm	EMG: 43.03 dB	^[^ ^115^ ^]^

^a)^ Modulus of the flexible substrate or electrode;

^b)^ Conv., conventional commercial Ag/AgCl electrodes.

Therefore, in order to increase the SNR or reduce measurement impedance, it is beneficial to maintain high capacitance and low resistance of the skin–electrode interfaces. On‐skin electrodes possess the unique features of conformal contact with skin, breathability to dissipate epidermis hydration, which result in desirable interface capacitance and resistance. Therefore, the on‐skin electrodes reported demonstrated comparable, if not better, performances to their conventional counterparts. In practice, it is observed that conformal contact with skin plays the most crucial role in determining the quality of EP signals, as loss of contact or presence of air gap typically leads to significantly decrease of signal magnitude or even unsuccessful signal capture. The conductivity of electrodes, on the other hand, is also important to achieve a high SNR, as low conductivity obviously contributes to higher interface impedance.

Among the six features listed in Figure 1, high stretchability and conductivity are recognized as basic desirable properties of on‐skin electrodes, as together they enable normal functionality under deformed conditions. The adoption of elastomer (as matrix to form composite or function as substrate) and structural design can improve stretchability of on‐skin electrodes, while metallic nanomaterials based composites usually demonstrate high conductivity due to superior high conductivity of metals. For instance, stretchable polymer substrate such as silicone‐based substrates,^[^ ^56^, ^200^ ^]^ textile‐based substrates,^[^ ^201^ ^]^ and protein‐based substrates^[^ ^202^ ^]^ had been investigated and elastic composites such as PDMS−ECCs,^[^ ^102^ ^]^ AgNWs–PDMS mixtures,^[^ ^103^ ^]^ and Ag‐PU nanocomposites^[^ ^101^ ^]^ had been synthesized. Regarding structural design, typical deformable structures such as filamentary serpentine,^[^ ^203^ ^]^ wrinkles,^[^ ^60^ ^]^ and kirigami^[^ ^204^ ^]^ have been implemented. With those strategies, on‐skin electrodes had achieved outstanding stretchability of over 400%.^[^ ^106^ ^]^ Meanwhile, metallic nanomaterial‐based electrodes had realized remarkable conductivity through the incorporation of AgNWs,^[^ ^205^, ^206^ ^]^ Au nanomesh,^[^ ^44^ ^]^ and Ag nanoparticles.^[^ ^207^ ^]^ Recent reports indicated that electrode materials could exhibited conductivity as high as 72600 S cm^−1^.^[^ ^104^ ^]^

Although significant progress has been made to improve the stretchability and conductivity, there are still certain challenges to be addressed to realize practical on‐skin electrodes of high performances. One challenge is tunable adhesion to skin. High level of adhesion to skin is critical to enhance low measurement impedance and mitigate the influence of motion artifacts. Therefore, adhesion enhancement strategies such as designing microstructures for dry adhesives (octopus‐inspired suckers,^[^ ^83^, ^125^, ^126^, ^127^ ^]^ clingfish‐inspired discs,^[^ ^128^ ^]^ gecko‐like surface,^[^ ^122^, ^124^ ^]^ mechanical interlocking,^[^ ^129^, ^130^ ^]^ cilia patterns,^[^ ^208^ ^]^ etc.) or adding biocompatible adhesives or pressure sensitive adhesives (silicon derivatives,^[^ ^56^ ^]^ Ca ions,^[^ ^121^ ^]^ PEIE,^[^ ^142^ ^]^ Triton X,^[^ ^103^ ^]^ etc.) have been investigated. Those approaches were fairly successful as lower electrode–skin contact impedance, compared with conventional Ag/AgCl electrodes, had been demonstrated. However, high adhesion can also be undesirable when the on‐skin electrode patch has to be peeled off after use, especially from wounded skin. Therefore, tunable adhesion is important to avoid skin damage and guarantee repeated use of on‐skin electrodes. However, the development of tunable adhesion is still rather scarce so far.

In addition, on‐skin electrodes with breathable properties are highly desired. The breathability of on‐skin electrodes is mainly achieved by designing nanomesh materials or adopting porous substrates so far.^[^ ^100^, ^209^, ^210^ ^]^ For instance, multiple types of porous substrates were successfully added to on‐skin electrodes (e.g., microperforated soft silicone,^[^ ^152^ ^]^ silicone elastomer sponges,^[^ ^86^ ^]^ textile^[^ ^211^ ^]^). Fabricated through electrospinning process, breathable electrodes with nanofiber scaffolds (e.g., PVDF nanofiber,^[^ ^212^ ^]^ AgNWs‐PA6 network^[^ ^100^ ^]^) were proved to be effective for air and vapor transmission. The WVTR of on‐skin electrodes reached as high as ≈18 g m^−2^ h^−1^. Meanwhile, emerging materials with active moisture wicking function have also been investigated (hygroscopic fibrous membranes^[^ ^213^ ^]^ and hygroscopic serpentine‐gel network^[^ ^210^ ^]^). Nevertheless, due to large numbers of voids in those breathable electrodes, it is difficult to maintain adhesion and mechanical durability simultaneously due to the reduction in contact areas with skin.

The power sources for standalone on‐skin electrode systems can be another challenging issue. It has been shown that the fully integrated on‐skin electrode systems can be energy demanding due to the integration of data processing and wireless transmission units.^[^ ^214^, ^215^, ^216^ ^]^ Moreover, there is a trend to integrate other types of sensors, such as temperature sensors,^[^ ^159^ ^]^ accelerometers,^[^ ^176^ ^]^ strain sensors,^[^ ^183^ ^]^ hydration sensors,^[^ ^200^ ^]^ tactile sensors,^[^ ^217^, ^218^ ^]^ etc., into on‐skin electrode systems for more comprehensive monitoring, which will clearly drive up the energy consumption. Thus, it is difficult to provide wearable and high energy density power sources for the integrated systems. Flexible supercapacitors were proposed but their applications are limited owing to low volumetric energy densities and small sizes.^[^ ^219^, ^220^ ^]^ NFC techniques represent another potential solution through inductive resonant energy transfer,^[^ ^181^, ^221^ ^]^ but NFC‐electrode systems must be close to the receiving terminal, not exceeding the maximum transmitter–receiver distance.^[^ ^222^ ^]^ Consequently, future research is not only needed to develop wearable power supply with high energy density but also to reduce power consumption of integrated electronic systems.

Scalable fabrication schemes of on‐skin electrodes with relative low costs are also very important to enable their practical applications. Conventional processes compatible with IC fabrication, including photolithographic patterning,^[^ ^61^ ^]^ etching,^[^ ^58^ ^]^ and electron beam deposition,^[^ ^118^ ^]^ can be economical solutions if high volume production is achieved. However, they are not directly compatible with flexible electronics manufacture, which involves soft substrate. Emerging manufacturing techniques, such as laser direct writing,^[^ ^223^, ^224^ ^]^ inkjet printing,^[^ ^225^, ^226^ ^]^ electrospraying,^[^ ^227^ ^]^ and electrospinning approach^[^ ^100^, ^228^ ^]^ can be valuable alternatives. Notably, the “cut and paste” process developed by Lu et al. represents an effective and highly efficient strategy for scalable fabrication of on‐skin electrodes.^[^ ^229^ ^]^

Due to superior properties of wearability and high measurement performances, on‐skin electrodes and their integrated systems have been successfully demonstrated in various applications. More specifically, they were used for disease diagnosis (e.g., dysphagia,^[^ ^189^ ^]^ arrhythmia,^[^ ^28^, ^230^ ^]^ epilepsy^[^ ^231^ ^]^) and emotion identification,^[^ ^232^ ^]^ gesture recognition,^[^ ^59^ ^]^ prosthetic control,^[^ ^152^ ^]^ driverless car,^[^ ^102^ ^]^ etc. Through multidisciplinary studies with machine learning and artificial intelligence, on‐skin electrode systems can be adopted for more sophisticated analysis and the measurement accuracy can be significantly increased.^[^ ^150^, ^164^ ^]^ Although there are still challenges, great progress has already been made in on‐skin electrodes for EP monitoring and HMI. With enormous efforts continuously invested on material synthesis and optimization, structural design, fabrication process, system integration and machine intelligence algorithms, we anticipate increasingly practical applications of on‐skin electrodes and systems.

## Conflict of Interest

The authors declare no conflict of interest.

## References

[1] C. Cea , G. D. Spyropoulos , P. Jastrzebska‐Perfect , J. J. Ferrero , J. N. Gelinas , D. Khodagholy , Nat. Mater. 2020, 19, 679. 32203456 10.1038/s41563-020-0638-3

[2] D. Son , J. Kang , O. Vardoulis , Y. Kim , N. Matsuhisa , J. Y. Oh , J. W. F. To , J. Mun , T. Katsumata , Y. Liu , A. F. McGuire , M. Krason , F. Molina‐Lopez , J. Ham , U. Kraft , Y. Lee , Y. Yun , J. B. H. Tok , Z. Bao , Nat. Nanotechnol. 2018, 13, 1057. 30127474 10.1038/s41565-018-0244-6

[3] J. Li , Y. Wang , L. Liu , S. Xu , Y. Liu , J. Leng , S. Cai , Adv. Funct. Mater. 2019, 29, 1903762.

[4] P. P. Vu , A. K. Vaskov , Z. T. Irwin , P. T. Henning , D. R. Lueders , A. T. Laidlaw , A. J. Davis , C. S. Nu , D. H. Gates , R. B. Gillespie , S. W. P. Kemp , T. A. Kung , C. A. Chestek , P. S. Cederna , Sci. Transl. Med. 2020, 12, eaay2857. 32132217 10.1126/scitranslmed.aay2857 PMC8082695

[5] R. A. Nawrocki , H. Jin , S. Lee , T. Yokota , M. Sekino , T. Someya , Adv. Funct. Mater. 2018, 28, 1803279.

[6] S. R. Soekadar , M. Witkowski , C. Gómez , E. Opisso , J. Medina , M. Cortese , M. Cempini , M. C. Carrozza , L. G. Cohen , N. Birbaumer , N. Vitiello , Sci. Rob. 2016, 1, eaag3296. 10.1126/scirobotics.aag3296 33157855

[7] B. J. Edelman , J. Meng , D. Suma , C. Zurn , E. Nagarajan , B. S. Baxter , C. C. Cline , B. He , Sci. Rob. 2019, 4, eaaw6844. 10.1126/scirobotics.aaw6844 PMC6814169 31656937

[8] H. U. Chung , A. Y. Rwei , A. Hourlier‐Fargette , S. Xu , K. Lee , E. C. Dunne , Z. Xie , C. Liu , A. Carlini , D. H. Kim , D. Ryu , E. Kulikova , J. Cao , I. C. Odland , K. B. Fields , B. Hopkins , A. Banks , C. Ogle , D. Grande , J. B. Park , J. Kim , M. Irie , H. Jang , J. Lee , Y. Park , J. Kim , H. H. Jo , H. Hahm , R. Avila , Y. Xu , M. Namkoong , J. W. Kwak , E. Suen , M. A. Paulus , R. J. Kim , B. V. Parsons , K. A. Human , S. S. Kim , M. Patel , W. Reuther , H. S. Kim , S. H. Lee , J. D. Leedle , Y. Yun , S. Rigali , T. Son , I. Jung , H. Arafa , V. R. Soundararajan , A. Ollech , A. Shukla , A. Bradley , M. Schau , C. M. Rand , L. E. Marsillio , Z. L. Harris , Y. Huang , A. Hamvas , A. S. Paller , D. E. Weese‐Mayer , J. Y. Lee , J. A. Rogers , Nat. Med. 2020, 26, 418. 32161411 10.1038/s41591-020-0792-9 PMC7315772

[9] S. Mishra , Y.‐S. Kim , J. Intarasirisawat , Y.‐T. Kwon , Y. Lee , M. Mahmood , H.‐R. Lim , R. Herbert , K. J. Yu , C. S. Ang , W. ‐H. Yeo , Sci. Adv. 2020, 6, eaay1729. 32201718 10.1126/sciadv.aay1729 PMC7069716

[10] X. Pu , H. Guo , J. Chen , X. Wang , Y. Xi , C. Hu , Z. L. Wang , Sci. Adv. 2017, 3, e1700694. 28782029 10.1126/sciadv.1700694 PMC5533541

[11] J. Van Cutsem , S. Marcora , K. De Pauw , S. Bailey , R. Meeusen , B. Roelands , Sports Med. 2017, 47, 1569. 28044281 10.1007/s40279-016-0672-0

[12] C. J. De Luca , J. Appl. Biomech. 1997, 13, 135.

[13] Y. Xu , B. Sun , Y. Ling , Q. Fei , Z. Chen , X. Li , P. Guo , N. Jeon , S. Goswami , Y. Liao , S. Ding , Q. Yu , J. Lin , G. Huang , Z. Yan , Proc. Natl. Acad. Sci. USA 2020, 117, 205. 31871158 10.1073/pnas.1917762116 PMC6955345

[14] S. Srinivasan , M. J. Carty , P. W. Calvaresi , T. R. Clites , B. E. Maimon , C. R. Taylor , A. N. Zorzos , H. Herr , Sci. Rob. 2017, 2, eaan2971. 10.1126/scirobotics.aan2971 33157872

[15] H. Hallez , B. Vanrumste , R. Grech , J. Muscat , W. De Clercq , A. Vergult , Y. D'Asseler , K. P. Camilleri , S. G. Fabri , S. Van Huffel , I. Lemahieu , J. Neuroeng. Rehabil. 2007, 4, 46. 18053144 10.1186/1743-0003-4-46 PMC2234413

[16] D. Khodagholy , J. N. Gelinas , Z. Zhao , M. Yeh , M. Long , J. D. Greenlee , W. Doyle , O. Devinsky , G. Buzsáki , Sci. Adv. 2016, 2, e1601027. 28861464 10.1126/sciadv.1601027 PMC5569954

[17] W. H. R. Miltner , C. Braun , M. Arnold , H. Witte , E. Taub , Nature 1999, 397, 434. 9989409 10.1038/17126

[18] Y. Ma , Y. Zhang , S. Cai , Z. Han , X. Liu , F. Wang , Y. Cao , Z. Wang , H. Li , Y. Chen , X. Feng , Adv. Mater. 2020, 32, 1902062. 10.1002/adma.201902062 31243834

[19] C. Wang , C. Wang , Z. Huang , S. Xu , Adv. Mater. 2018, 30, 1801368. 10.1002/adma.201801368 30073715

[20] Y. Liu , K. He , G. Chen , W. R. Leow , X. Chen , Chem. Rev. 2017, 117, 12893. 28991450 10.1021/acs.chemrev.7b00291

[21] S. P. Lacour , G. Courtine , J. Guck , Nat. Rev. Mater. 2016, 1, 16063.

[22] J. C. Yang , J. Mun , S. Y. Kwon , S. Park , Z. Bao , S. Park , Adv. Mater. 2019, 31, 1904765. 10.1002/adma.201904765 31538370

[23] W. Gao , H. Ota , D. Kiriya , K. Takei , A. Javey , Acc. Chem. Res. 2019, 52, 523. 30767497 10.1021/acs.accounts.8b00500

[24] H. Wu , Y. A. Huang , F. Xu , Y. Q. Duan , Z. P. Yin , Adv. Mater. 2016, 28, 9881. 27677428 10.1002/adma.201602251

[25] K.‐I. Jang , H. U. Chung , S. Xu , C. H. Lee , H. Luan , J. Jeong , H. Cheng , G.‐T. Kim , S. Y. Han , J. W. Lee , J. Kim , M. Cho , F. Miao , Y. Yang , H. N. Jung , M. Flavin , H. Liu , G. W. Kong , K. J. Yu , S. I. Rhee , J. Chung , B. Kim , J. W. Kwak , M. H. Yun , J. Y. Kim , Y. M. Song , U. Paik , Y. Zhang , Y. Huang , J. A. Rogers , Nat. Commun. 2015, 6, 6566. 25782446 10.1038/ncomms7566 PMC4383007

[26] S. Choi , H. Lee , R. Ghaffari , T. Hyeon , D. H. Kim , Adv. Mater. 2016, 28, 4203. 26779680 10.1002/adma.201504150

[27] W. Wu , H. Haick , Adv. Mater. 2018, 30, 1705024. 10.1002/adma.201705024 29498115

[28] Y.‐S. Kim , M. Mahmood , Y. Lee , N. K. Kim , S. Kwon , R. Herbert , D. Kim , H. C. Cho , W.‐H. Yeo , Adv. Sci. 2019, 6, 1900939. 10.1002/advs.201900939 PMC6724359 31508289

[29] H. Jin , N. Matsuhisa , S. Lee , M. Abbas , T. Yokota , T. Someya , Adv. Mater. 2017, 29, 1605848. 10.1002/adma.201605848 28370661

[30] M. Tang , P. Zheng , K. Wang , Y. Qin , Y. Jiang , Y. Cheng , Z. Li , L. Wu , J. Mater. Chem. A 2019, 7, 27278.

[31] Q. Wang , S. Ling , X. Liang , H. Wang , H. Lu , Y. Zhang , Adv. Funct. Mater. 2019, 29, 1808695.

[32] T.‐P. Huynh , P. Sonar , H. Haick , Adv. Mater. 2017, 29, 1604973. 10.1002/adma.201604973 28229499

[33] E. Huigen , A. Peper , C. A. Grimbergen , Med. Biol. Eng. Comput. 2002, 40, 332. 12195981 10.1007/BF02344216

[34] J.‐H. Ahn , J. H. Je , J. Phys. D: Appl. Phys. 2012, 45, 103001.

[35] J. A. Rogers , T. Someya , Y. Huang , Science 2010, 327, 1603. 20339064 10.1126/science.1182383

[36] B. Yu , S.‐Y. Kang , A. Akthakul , N. Ramadurai , M. Pilkenton , A. Patel , A. Nashat , D. G. Anderson , F. H. Sakamoto , B. A. Gilchrest , R. R. Anderson , R. Langer , Nat. Mater. 2016, 15, 911. 27159017 10.1038/nmat4635

[37] A. J. Gallagher , A. Ní Annaidh , K. Bruyère , M. Otténio , H. Xie , M. D. Gilchrist , IRCOBI Conf. 2012, 40, 494.

[38] V. Arumugam , M. Naresh , R. Sanjeevi , J. Biosci. 1994, 19, 307.

[39] X. Chen , Small Methods 2017, 1, 1600029.

[40] A. N. Annaidh , K. Bruyère , M. Destrade , M. D. Gilchrist , M. Otténio , J. Mech. Behav. Biomed. Mater. 2012, 5, 139. 22100088 10.1016/j.jmbbm.2011.08.016

[41] S. Baik , H. J. Lee , D. W. Kim , J. W. Kim , Y. Lee , C. Pang , Adv. Mater. 2019, 31, 1803309. 10.1002/adma.201803309 30773697

[42] I. Hwang , H. N. Kim , M. Seong , S.‐H. Lee , M. Kang , H. Yi , W. G. Bae , M. K. Kwak , H. E. Jeong , Adv. Healthcare Mater. 2018, 7, 1800275. 10.1002/adhm.201800275 29757494

[43] S. Luebberding , N. Krueger , M. Kerscher , Int. J. Cosmet. Sci. 2013, 35, 477. 23713991 10.1111/ics.12068

[44] A. Miyamoto , S. Lee , N. F. Cooray , S. Lee , M. Mori , N. Matsuhisa , H. Jin , L. Yoda , T. Yokota , A. Itoh , M. Sekino , H. Kawasaki , T. Ebihara , M. Amagai , T. Someya , Nat. Nanotechnol. 2017, 12, 907. 28737748 10.1038/nnano.2017.125

[45] M. A. Yokus , J. S. Jur , IEEE Trans. Biomed. Eng. 2015, 63, 423. 26241969 10.1109/TBME.2015.2462312

[46] J. Visser , F. P. Melchels , J. E. Jeon , E. M. Van Bussel , L. S. Kimpton , H. M. Byrne , W. J. Dhert , P. D. Dalton , D. W. Hutmacher , J. Malda , Nat. Commun. 2015, 6, 6933. 25917746 10.1038/ncomms7933

[47] J. Rivnay , R. M. Owens , G. G. Malliaras , Chem. Mater. 2014, 26, 679.

[48] S. K. Ameri , P. Singh , S. Sonkusale , Biosens. Bioelectron. 2014, 61, 625. 24967752 10.1016/j.bios.2014.05.067

[49] L. Z. Xu , S. R. Gutbrod , A. P. Bonifas , Y. W. Su , M. S. Sulkin , N. S. Lu , H. J. Chung , K. I. Jang , Z. J. Liu , M. Ying , C. Lu , R. C. Webb , J. S. Kim , J. I. Laughner , H. Y. Cheng , Y. H. Liu , A. Ameen , J. W. Jeong , G. T. Kim , Y. G. Huang , I. R. Efimov , J. A. Rogers , Nat. Commun. 2014, 5, 3329. 24569383 10.1038/ncomms4329 PMC4521772

[50] L. Guo , G. S. Guvanasen , X. Liu , C. Tuthill , T. R. Nichols , S. P. DeWeerth , IEEE Trans. Biomed. Circ. Syst. 2013, 7, 1. 10.1109/TBCAS.2012.2192932 23853274

[51] Z. Zhao , L. Luan , X. Wei , H. Zhu , X. Li , S. Lin , J. J. Siegel , R. A. Chitwood , C. Xie , Nano Lett. 2017, 17, 4588. 28682082 10.1021/acs.nanolett.7b00956 PMC5869028

[52] L. Ren , S. Xu , J. Gao , Z. Lin , Z. Chen , B. Liu , L. Liang , L. Jiang , Sensors 2018, 18, 1191. 10.3390/s18041191 PMC5948552 29652835

[53] Y. Meng , Z. Li , J. Chen , Microsyst. Technol. 2016, 22, 2027.

[54] J. E. Carsley , A. Fisher , W. W. Milligan , E. C. Aifantis , Metall. Mater. Trans. A 1998, 29, 2261.

[55] B. X. Xu , A. Akhtar , Y. H. Liu , H. Chen , W. H. Yeo , S. I. I. Park , B. Boyce , H. Kim , J. W. Yu , H. Y. Lai , S. Y. Jung , Y. H. Zhou , J. Kim , S. Cho , Y. G. Huang , T. Bretl , J. A. Rogers , Adv. Mater. 2016, 28, 4462. 26469201 10.1002/adma.201504155 PMC4833675

[56] K. I. Jang , S. Y. Han , S. Xu , K. E. Mathewson , Y. H. Zhang , J. W. Jeong , G. T. Kim , C. Webb , J. W. Lee , T. J. Dawidczyk , R. H. Kim , Y. M. Song , W. H. Yeo , S. Kim , H. Y. Cheng , S. Il Rhee , J. Chung , B. Kim , H. U. Chung , D. J. Lee , Y. Y. Yang , M. Cho , J. G. Gaspar , R. Carbonari , M. Fabiani , G. Gratton , Y. G. Huang , J. A. Rogers , Nat. Commun. 2014, 5, 4779. 25182939 10.1038/ncomms5779

[57] D.‐H. Kim , N. Lu , R. Ma , Y.‐S. Kim , R.‐H. Kim , S. Wang , J. Wu , S. M. Won , H. Tao , A. Islam , K. J. Yu , T.‐i. Kim , R. Chowdhury , M. Ying , L. Xu , M. Li , H.‐J. Chung , H. Keum , M. McCormick , P. Liu , Y.‐W. Zhang , F. G. Omenetto , Y. Huang , T. Coleman , J. A. Rogers , Science 2011, 333, 838. 21836009 10.1126/science.1206157

[58] J. J. S. Norton , D. S. Lee , J. W. Lee , W. Lee , O. Kwon , P. Won , S. Y. Jung , H. Y. Cheng , J. W. Jeong , A. Akce , S. Umunna , I. Na , Y. H. Kwon , X. Q. Wang , Z. J. Liu , U. Paik , Y. G. Huang , T. Bretl , W. H. Yeo , J. A. Rogers , Proc. Natl. Acad. Sci. USA 2015, 112, 3920. 25775550 10.1073/pnas.1424875112 PMC4386388

[59] N. Kim , T. Lim , K. Song , S. Yang , J. Lee , ACS Appl. Mater. Interfaces 2016, 8, 21070. 27500864 10.1021/acsami.6b05025

[60] G. Chen , N. Matsuhisa , Z. Liu , D. Qi , P. Cai , Y. Jiang , C. Wan , Y. Cui , W. R. Leow , Z. Liu , S. Gong , K.‐Q. Zhang , Y. Cheng , X. Chen , Adv. Mater. 2018, 30, 1800129. 10.1002/adma.201800129 29603437

[61] J. W. Jeong , W. H. Yeo , A. Akhtar , J. J. S. Norton , Y. J. Kwack , S. Li , S. Y. Jung , Y. W. Su , W. Lee , J. Xia , H. Y. Cheng , Y. G. Huang , W. S. Choi , T. Bretl , J. A. Rogers , Adv. Mater. 2013, 25, 6839. 24327417 10.1002/adma.201301921

[62] F. Torres , M. das Graças , M. Melo , A. Tosti , Clin. Cosmet. Invest. Dermatol. 2009, 2, 39. 10.2147/ccid.s3693 PMC3047925 21436967

[63] S. M. Lee , J. H. Kim , H. J. Byeon , Y. Y. Choi , K. S. Park , S. H. Lee , J. Neural Eng. 2013, 10, 036006. 23574793 10.1088/1741-2560/10/3/036006

[64] J. W. Jeong , M. K. Kim , H. Y. Cheng , W. H. Yeo , X. Huang , Y. H. Liu , Y. H. Zhang , Y. G. Huang , J. A. Rogers , Adv. Healthcare Mater. 2014, 3, 642. 10.1002/adhm.201300334 24132942

[65] Q. Huang , Y. Zhu , Adv. Mater. Technol. 2019, 4, 1800546.

[66] S. X. Yang , Y. C. Chen , L. Nicolini , P. Pasupathy , J. Sacks , B. Su , R. Yang , D. Sanchez , Y. F. Chang , P. L. Wang , D. Schnyer , D. Neikirk , N. S. Lu , Adv. Mater. 2015, 27, 6423. 26398335 10.1002/adma.201502386

[67] T. Ha , J. Tran , S. Y. Liu , H. Jang , H. Jeong , R. Mitbander , H. Huh , Y. T. Qiu , J. Duong , R. L. Wang , P. L. Wang , A. Tandon , J. Sirohi , N. S. Lu , Adv. Sci. 2019, 6, 1900290. 10.1002/advs.201900290 PMC6662084 31380208

[68] C. R. Guo , Y. Yu , J. Liu , J. Mater. Chem. B 2014, 2, 5739. 32262017 10.1039/c4tb00660g

[69] S. S. Yao , Y. Zhu , JOM 2016, 68, 1145.

[70] M. Miao , Carbon 2011, 49, 3755.

[71] Z. Han , A. Fina , Prog. Polym. Sci. 2011, 36, 914.

[72] H. Jung , J. Moon , D. Baek , J. Lee , Y. Choi , J. Hong , S. Lee , IEEE Trans. Biomed. Eng. 2012, 59, 1472. 22410324 10.1109/TBME.2012.2190288

[73] B. Y. Liu , Y. M. Chen , Z. Y. Luo , W. Z. Zhang , Q. Tu , X. Jin , J. Biomater. Sci., Polym. Ed. 2015, 26, 1229. 26268887 10.1080/09205063.2015.1082807

[74] B. Y. Liu , Z. Y. Luo , W. Z. Zhang , Q. Tu , X. Jin , J. Biomater. Sci., Polym. Ed. 2016, 27, 1899. 27659794 10.1080/09205063.2016.1239951

[75] S. M. Lee , H. J. Byeon , J. H. Lee , D. H. Baek , K. H. Lee , J. S. Hong , S. H. Lee , Sci. Rep. 2015, 4, 6074. 10.1038/srep06074 PMC4133715 25123356

[76] T. Kim , J. Park , J. Sohn , D. Cho , S. Jeon , ACS Nano 2016, 10, 4770. 26986477 10.1021/acsnano.6b01355

[77] M. Martin‐Gallego , M. M. Bernal , M. Hernandez , R. Verdejo , M. A. López‐Manchado , Eur. Polym. J. 2013, 49, 1347.

[78] J. Du , L. Zhao , Y. Zeng , L. Zhang , F. Li , P. Liu , C. Liu , Carbon 2011, 49, 1094.

[79] K. Kostarelos , M. Vincent , C. Hebert , J. A. Garrido , Adv. Mater. 2017, 29, 1700909. 10.1002/adma.201700909 28901588

[80] X. Du , L. Wu , J. Cheng , S. Huang , Q. Cai , Q. Jin , J. Zhao , J. Biol. Phys. 2015, 41, 339. 25712492 10.1007/s10867-015-9382-3 PMC4550624

[81] D.‐W. Park , A. A. Schendel , S. Mikael , S. K. Brodnick , T. J. Richner , J. P. Ness , M. R. Hayat , F. Atry , S. T. Frye , R. Pashaie , S. Thongpang , Z. Ma , J. C. Williams , Nat. Commun. 2014, 5, 5258. 25327513 10.1038/ncomms6258 PMC4218963

[82] S. Kabiri Ameri , R. Ho , H. Jang , L. Tao , Y. Wang , L. Wang , D. M. Schnyer , D. Akinwande , N. Lu , ACS Nano 2017, 11, 7634. 28719739 10.1021/acsnano.7b02182

[83] S. Chun , W. Son , D. W. Kim , J. Lee , H. Min , H. Jung , D. Kwon , A. H. Kim , Y.‐J. Kim , S. K. Lim , C. Pang , C. Choi , ACS Appl. Mater. Interfaces 2019, 11, 16951. 31034198 10.1021/acsami.9b04206

[84] M. K. Yapici , A. A. Tamador , Y. A. Samad , K. Liao , Sens. Actuators, B 2015, 221, 1469.

[85] Z. Li , W. Guo , Y. Huang , K. Zhu , H. Yi , H. Wu , Carbon 2020, 164, 164.

[86] B. Sun , R. N. McCay , S. Goswami , Y. Xu , C. Zhang , Y. Ling , J. Lin , Z. Yan , Adv. Mater. 2018, 30, 1804327. 10.1002/adma.201804327 30306662

[87] S. Gong , W. Schwalb , Y. Wang , Y. Chen , Y. Tang , J. Si , B. Shirinzadeh , W. Cheng , Nat. Commun. 2014, 5, 3132. 24495897 10.1038/ncomms4132

[88] T. Wang , R. Wang , Y. Cheng , J. Sun , ACS Appl. Mater. Interfaces 2016, 8, 9297. 26895474 10.1021/acsami.5b11143

[89] Z. B. Yu , Q. W. Zhang , L. Li , Q. Chen , X. F. Niu , J. Liu , Q. B. Pei , Adv. Mater. 2011, 23, 664. 21274917 10.1002/adma.201003398

[90] H. J. Kim , S. H. Lee , J. Lee , E. S. Lee , J. H. Choi , J. H. Jung , J. Y. Jung , D. G. Choi , Small 2014, 10, 3767. 24840606 10.1002/smll.201400911

[91] H. Y. Jang , S.‐K. Lee , S. H. Cho , J.‐H. Ahn , S. Park , Chem. Mater. 2013, 25, 3535.

[92] C. F. Guo , T. Y. Sun , Q. H. Liu , Z. G. Suo , Z. F. Ren , Nat. Commun. 2014, 5, 3121. 24469072 10.1038/ncomms4121

[93] M. Su , F. Li , S. Chen , Z. Huang , M. Qin , W. Li , X. Zhang , Y. Song , Adv. Mater. 2016, 28, 1369. 26644086 10.1002/adma.201504759

[94] R. M. Yu , W. Z. Wu , Y. Ding , Z. L. Wang , ACS Nano 2013, 7, 6403. 23777447 10.1021/nn4026788

[95] A. C. Myers , H. Huang , Y. Zhu , RSC Adv. 2015, 5, 11627.

[96] Y. Zhu , Q. Qin , F. Xu , F. Fan , Y. Ding , T. Zhang , B. J. Wiley , Z. L. Wang , Phys. Rev. B 2012, 85, 045443.

[97] K. J. Seo , Y. Qiang , I. Bilgin , S. Kar , C. Vinegoni , R. Weissleder , H. Fang , ACS Nano 2017, 11, 4365. 28391679 10.1021/acsnano.7b01995

[98] S. Han , M. K. Kim , B. Wang , D. S. Wie , S. D. Wang , C. H. Lee , Adv. Mater. 2016, 28, 10257. 27714861 10.1002/adma.201603878

[99] Y. J. Fan , X. Li , S. Y. Kuang , Y. Kuang , L. Zhang , Y. H. Chen , L. Liu , K. Zhang , S. W. Ma , F. Liang , T. Wu , Z. L. Wang , G. Zhu , ACS Nano 2018, 12, 9326. 30118595 10.1021/acsnano.8b04245

[100] L. Liu , H. Y. Li , Y. J. Fan , Y. H. Chen , S. Y. Kuang , Z. B. Li , Z. L. Wang , G. Zhu , Small 2019, 15, 1900755. 10.1002/smll.201900755 31021507

[101] Z. Jiang , M. O. G. Nayeem , K. Fukuda , S. Ding , H. Jin , T. Yokota , D. Inoue , D. Hashizume , T. Someya , Adv. Mater. 2019, 31, 1903446. 10.1002/adma.201903446 31339196

[102] W. Guo , P. Zheng , X. Huang , H. Zhuo , Y. Wu , Z. Yin , Z. Li , H. Wu , ACS Appl. Mater. Interfaces 2019, 11, 8567. 30729786 10.1021/acsami.8b21836

[103] J.‐H. Kim , S.‐R. Kim , H.‐J. Kil , Y.‐C. Kim , J.‐W. Park , Nano Lett. 2018, 18, 4531. 29923729 10.1021/acs.nanolett.8b01743

[104] S. Choi , S. I. Han , D. Jung , H. J. Hwang , C. Lim , S. Bae , O. K. Park , C. M. Tschabrunn , M. Lee , S. Y. Bae , J. W. Yu , J. H. Ryu , S. W. Lee , K. Park , P. M. Kang , W. B. Lee , R. Nezafat , T. Hyeon , D. H. Kim , Nat. Nanotechnol. 2018, 13, 1048. 30104619 10.1038/s41565-018-0226-8

[105] D. P. Qi , Z. Y. Liu , M. Yu , Y. Liu , Y. X. Tang , J. H. Lv , Y. C. Li , J. Wei , L. Bo , Z. Yu , X. D. Chen , Adv. Mater. 2015, 27, 3145. 25865755 10.1002/adma.201405807

[106] P. Won , J. J. Park , T. Lee , I. Ha , S. Han , M. Choi , J. Lee , S. Hong , K.‐J. Cho , S. H. Ko , Nano Lett. 2019, 19, 6087. 31411037 10.1021/acs.nanolett.9b02014

[107] Y. Khan , F. J. Pavinatto , M. C. Lin , A. Liao , S. L. Swisher , K. Mann , V. Subramanian , M. M. Maharbiz , A. C. Arias , Adv. Funct. Mater. 2016, 26, 1004.

[108] S. Venkatraman , J. Hendricks , Z. A. King , A. J. Sereno , S. Richardson‐Burns , D. Martin , J. M. Carmena , IEEE Trans. Neural Syst. Rehabil. Eng. 2011, 19, 307. 21292598 10.1109/TNSRE.2011.2109399

[109] T. Sekitani , T. Yokota , K. Kuribara , M. Kaltenbrunner , T. Fukushima , Y. Inoue , M. Sekino , T. Isoyama , Y. Abe , H. Onodera , T. Someya , Nat. Commun. 2016, 7, 11425. 27125910 10.1038/ncomms11425 PMC5411732

[110] M. Berggren , A. Richter‐Dahlfors , Adv. Mater. 2007, 19, 3201.

[111] P. Leleux , J. M. Badier , J. Rivnay , C. Benar , T. Herve , P. Chauvel , G. G. Malliaras , Adv. Healthcare Mater. 2014, 3, 490. 10.1002/adhm.201300311 24106008

[112] E. Bihar , T. Roberts , M. Saadaoui , T. Herve , J. B. De Graaf , G. G. Malliaras , Adv. Healthcare Mater. 2017, 6, 1601167. 10.1002/adhm.201601167 28121395

[113] C. Xie , X. Wang , H. He , Y. Ding , X. Lu , Adv. Funct. Mater. 2020, 30, 1909954.

[114] K. Nagamine , S. Chihara , H. Kai , H. Kaji , M. Nishizawa , Sens. Actuators, B 2016, 237, 49.

[115] S. Pan , F. Zhang , P. Cai , M. Wang , K. He , Y. Luo , Z. Li , G. Chen , S. Ji , Z. Liu , X. J. Loh , X. Chen , Adv. Funct. Mater. 2020, 30, 1909540.

[116] P. A. Lopes , D. V. Gomes , D. G. Marques , P. Faia , J. Gis , T. F. Patrcio , J. Coelho , A. Serra , A. T. de Almeida , C. Majidi , M. Tavakoli , Adv. Healthcare Mater. 2019, 8, 1900234. 10.1002/adhm.201900234 31273945

[117] X. Huang , Y. Liu , K. Chen , W. J. Shin , C. J. Lu , G. W. Kong , D. Patnaik , S. H. Lee , J. F. Cortes , J. A. Rogers , Small 2014, 10, 3083. 24706477 10.1002/smll.201400483

[118] W. H. Yeo , Y. S. Kim , J. Lee , A. Ameen , L. K. Shi , M. Li , S. D. Wang , R. Ma , S. H. Jin , Z. Kang , Y. G. Huang , J. A. Rogers , Adv. Mater. 2013, 25, 2773. 23440975 10.1002/adma.201204426

[119] J. Kim , A. Banks , H. Cheng , Z. Xie , S. Xu , I. K. Jang , J. W. Lee , Z. Liu , P. Gutruf , X. Huang , P. Wei , F. Liu , K. Li , M. Dalal , R. Ghaffari , X. Feng , Y. Huang , S. Gupta , U. Paik , J. A. Rogers , Small 2015, 11, 906. 25367846 10.1002/smll.201402495

[120] S. Lee , Y. Inoue , D. Kim , A. Reuveny , K. Kuribara , T. Yokota , J. Reeder , M. Sekino , T. Sekitani , Y. Abe , T. Someya , Nat. Commun. 2014, 5, 5898. 25523614 10.1038/ncomms6898

[121] J.‐W. Seo , H. Kim , K. Kim , S. Q. Choi , H. J. Lee , Adv. Funct. Mater. 2018, 28, 1800802.

[122] M. K. Kwak , H. E. Jeong , K. Y. Suh , Adv. Mater. 2011, 23, 3949. 21796686 10.1002/adma.201101694

[123] W. G. Bae , D. Kim , M. K. Kwak , L. Ha , S. M. Kang , K. Y. Suh , Adv. Healthcare Mater. 2013, 2, 109. 10.1002/adhm.201200098 23184425

[124] E. Kizilkan , S. N. Gorb , ACS Appl. Mater. Interfaces 2018, 10, 26752. 30010312 10.1021/acsami.8b06686

[125] S. Baik , Y. Park , T.‐J. Lee , S. H. Bhang , C. Pang , Nature 2017, 546, 396. 28617467 10.1038/nature22382

[126] S. Baik , J. Kim , H. J. Lee , T. H. Lee , C. Pang , Adv. Sci. 2018, 5, 1800100. 10.1002/advs.201800100 PMC6097001 30128235

[127] S. Chun , D. W. Kim , S. Baik , H. J. Lee , J. H. Lee , S. H. Bhang , C. Pang , Adv. Funct. Mater. 2018, 28, 1805224.

[128] P. Rao , T. L. Sun , L. Chen , R. Takahashi , G. Shinohara , H. Guo , D. R. King , T. Kurokawa , J. P. Gong , Adv. Mater. 2018, 30, 1801884. 10.1002/adma.201801884 29939425

[129] Z. Y. Liu , X. T. Wang , D. P. Qi , C. Xu , J. C. Yu , Y. Q. Liu , Y. Jiang , B. Liedberg , X. D. Chen , Adv. Mater. 2017, 29, 1603382. 10.1002/adma.201603382 27809367

[130] S. Y. Yang , E. D. O'Cearbhaill , G. C. Sisk , K. M. Park , W. K. Cho , M. Villiger , B. E. Bouma , B. Pomahac , J. M. Karp , Nat. Commun. 2013, 4, 1702. 23591869 10.1038/ncomms2715 PMC3660066

[131] J. Kenneth Langstreth , K. Kevin , A. D. Roberts , T. David , Proc. R. Soc. London, Ser. A. 1971, 324, 301.

[132] A. Jagota , C.‐Y. Hui , Mater. Sci. Eng., R 2011, 72, 253.

[133] L. F. Boesel , C. Greiner , E. Arzt , A. Del Campo , Adv. Mater. 2010, 22, 2125. 20349430 10.1002/adma.200903200

[134] M. Lamblet , E. Verneuil , T. Vilmin , A. Buguin , P. Silberzan , L. Leger , Langmuir 2007, 23, 6966. 17511481 10.1021/la063104h

[135] D. W. Kim , S. Baik , H. Min , S. Chun , H. J. Lee , K. H. Kim , J. Y. Lee , C. Pang , Adv. Funct. Mater. 2019, 29, 1807614.

[136] M. F. Tse , L. Jacob , J. Adhes. 1996, 56, 79.

[137] E. P. Chang , J. Adhes. 1997, 60, 233.

[138] S. Sun , M. Li , A. Liu , Int. J. Adhes. Adhes. 2013, 41, 98.

[139] B. K. Lee , J. H. Ryu , I. B. Baek , Y. Kim , W. I. Jang , S. H. Kim , Y. S. Yoon , S. H. Kim , S. G. Hong , S. Byun , H. Y. Yu , Adv. Healthcare Mater. 2017, 6, 1700621. 10.1002/adhm.201700621 28795496

[140] S. H. Jeong , S. Zhang , K. Hjort , J. Hilborn , Z. Wu , Adv. Mater. 2016, 28, 5830. 27167137 10.1002/adma.201505372

[141] J. Kim , Y Hwang , S. Jeong , S. Y. Lee , Y. Choi , S. Jung , J. Mater. Chem. C 2018, 6, 2210.

[142] Y. Yamamoto , D. Yamamoto , M. Takada , H. Naito , T. Arie , S. Akita , K. Takei , Adv. Healthcare Mater. 2017, 6, 1700495. 10.1002/adhm.201700495 28661047

[143] T. J. Widman , H. Oostman , F. J. Storrs , Dermatitis 2008, 19, 32. 18346394

[144] L. Xue , A. Kovalev , K. Dening , A. Eichler‐Volf , H. Eickmeier , M. Haase , D. Enke , M. Steinhart , S. N. Gorb , Nano Lett. 2013, 13, 5541. 24171547 10.1021/nl403144w

[145] H.‐H. Park , M. Seong , K. Sun , H. Ko , S. M. Kim , H. E. Jeong , ACS Macro Lett. 2017, 6, 1325. 10.1021/acsmacrolett.7b00829 35650811

[146] H. Lee , D. S. Um , Y. Lee , S. Lim , H. J. Kim , H. Ko , Adv. Mater. 2016, 28, 7457. 27322886 10.1002/adma.201601407

[147] D.‐M. Drotlef , P. Blümler , A. del Campo , Adv. Mater. 2014, 26, 775. 24259374 10.1002/adma.201303087

[148] M. J. Vogel , P. H. Steen , Proc. Natl. Acad. Sci. USA 2010, 107, 3377. 20133725 10.1073/pnas.0914720107 PMC2840443

[149] C. Heinzmann , S. Coulibaly , A. Roulin , G. L. Fiore , C. Weder , ACS Appl. Mater. Interfaces 2014, 6, 4713. 24484360 10.1021/am405302z

[150] Y. Zhang , T. H. Tao , Adv. Mater. 2019, 31, 1905767. 10.1002/adma.201905767 31621959

[151] Y. Zhao , H. Wang , H. Zhou , T. Lin , Small 2017, 13, 1601070. 10.1002/smll.201601070 27717131

[152] L. Tian , B. Zimmerman , A. Akhtar , K. J. Yu , M. Moore , J. Wu , R. J. Larsen , J. W. Lee , J. Li , Y. Liu , B. Metzger , S. Qu , X. Guo , K. E. Mathewson , J. A. Fan , J. Cornman , M. Fatina , Z. Xie , Y. Ma , J. Zhang , Y. Zhang , F. Dolcos , M. Fabiani , G. Gratton , T. Bretl , L. J. Hargrove , P. V. Braun , Y. Huang , J. A. Rogers , Nat. Biomed. Eng. 2019, 3, 194. 30948811 10.1038/s41551-019-0347-x

[153] W. Zhou , S. Yao , H. Wang , Q. Du , Y. Ma , Y. Zhu , ACS Nano 2020, 14, 5798. 32347707 10.1021/acsnano.0c00906

[154] K. A. McCulloh , J. S. Sperry , F. R. Adler , Nature 2003, 421, 939. 12607000 10.1038/nature01444

[155] Y. Dong , J. Kong , S. L. Phua , C. Zhao , N. L. Thomas , X. Lu , ACS Appl. Mater. Interfaces 2014, 6, 14087. 25020135 10.1021/am503417w

[156] H. Zhou , H. X. Wang , H. T. Niu , T. Lin , Sci. Rep. 2013, 3, 2964. 24129357 10.1038/srep02964 PMC3797433

[157] P.‐G. d. Gennes , F. Brochard‐Wyart , D. Quere , Capillarity and Wetting Phenomena: Drops, Bubbles, Pearls, Waves., 1st ed., Springer, Berlin 2004.

[158] J. Wu , N. Wang , L. Wang , H. Dong , Y. Zhao , L. Jiang , Soft Matter 2012, 8, 5996.

[159] S. Xu , Y. Zhang , L. Jia , K. E. Mathewson , K. I. Jang , J. Kim , H. Fu , X. Huang , P. Chava , R. Wang , S. Bhole , L. Wang , Y. J. Na , Y. Guan , M. Flavin , Z. Han , Y. Huang , J. A. Rogers , Science 2014, 344, 70. 24700852 10.1126/science.1250169

[160] K. I. Jang , K. Li , H. U. Chung , S. Xu , H. N. Jung , Y. Yang , J. W. Kwak , H. H. Jung , J. Song , C. Yang , A. Wang , Z. Liu , J. Y. Lee , B. H. Kim , J. H. Kim , J. Lee , Y. Yu , B. J. Kim , H. Jang , K. J. Yu , J. Kim , J. W. Lee , J. W. Jeong , Y. M. Song , Y. Huang , Y. Zhang , J. A. Rogers , Nat. Commun. 2017, 8, 15894. 28635956 10.1038/ncomms15894 PMC5482057

[161] J. W. Lee , R. Xu , S. Lee , K. I. Jang , Y. Yang , A. Banks , K. J. Yu , J. Kim , S. Xu , S. Ma , S. W. Jang , P. Won , Y. Li , B. H. Kim , J. Y. Choe , S. Huh , Y. H. Kwon , Y. Huang , U. Paik , J. A. Rogers , Proc. Natl. Acad. Sci. USA 2016, 113, 6131. 27185907 10.1073/pnas.1605720113 PMC4896718

[162] C. H. Lee , Y. Ma , K.‐I. Jang , A. Banks , T. Pan , X. Feng , J. S. Kim , D. Kang , M. S. Raj , B. L. McGrane , B. Morey , X. Wang , R. Ghaffari , Y. Huang , J. A. Rogers , Adv. Funct. Mater. 2015, 25, 3698.

[163] S. Lin , J. C. Liu , W. Z. Li , D. Wang , Y. Huang , C. Jia , Z. W. Li , M. Murtaza , H. Y. Wang , J. N. Song , Z. L. Liu , K. Huang , D. Zu , M. Lei , B. Hong , H. Wu , Nano Lett. 2019, 19, 6853. 31454250 10.1021/acs.nanolett.9b02019

[164] Z. Zeng , Z. Huang , K. Leng , W. Han , H. Niu , Y. Yu , Q. Ling , J. Liu , Z. Wu , J. Zang , ACS Sens. 2020. 10.1021/acssensors.9b02451 31939287

[165] H. Jeong , L. Wang , T. Ha , R. Mitbander , X. Yang , Z. Dai , S. Qiao , L. Shen , N. Sun , N. Lu , Adv. Mater. Technol. 2019, 4, 1900117.

[166] M. A. Mañanas , M. Rojas‐Martínez , J. F. Alonso , Clin. Neurophysiol. 2016, 127, 2532. 27117986 10.1016/j.clinph.2016.02.005

[167] F. V. Tenore , A. Ramos , A. Fahmy , S. Acharya , N. V. Thakor , IEEE Trans. Biomed. Eng. 2009, 56, 1427. 19473933 10.1109/TBME.2008.2005485

[168] M. Rojas‐Martínez , M. A. Mañanas , J. F. Alonso , R. Merletti , J. Electromyogr. Kinesiol. 2013, 23, 33. 22819519 10.1016/j.jelekin.2012.06.009

[169] M. Rojas‐Martínez , M. A. Mañanas , J. F. Alonso , J. Neuroeng. Rehabil. 2012, 9, 85. 23216679 10.1186/1743-0003-9-85 PMC3575258

[170] C. Chen , Y. Yu , S. Ma , X. Sheng , C. Lin , D. Farina , X. Zhu , Biomed. Signal. Process 2020, 55, 101637.

[171] E. Martinez‐Valdes , C. M. Laine , D. Falla , F. Mayer , D. Farina , Clin. Neurophysiol. 2016, 127, 2534. 26778718 10.1016/j.clinph.2015.10.065

[172] A. Stango , F. Negro , D. Farina , IEEE Trans. Neural Syst. Rehabil. Eng. 2014, 23, 189. 25389242 10.1109/TNSRE.2014.2366752

[173] S. Yao , P. Ren , R. Song , Y. Liu , Q. Huang , J. Dong , B. T. O'Connor , Y. Zhu , Adv. Mater. 2019, 32, 1902343. 10.1002/adma.201902343 31464046

[174] W. Gao , S. Emaminejad , H. Y. Y. Nyein , S. Challa , K. Chen , A. Peck , H. M. Fahad , H. Ota , H. Shiraki , D. Kiriya , D.‐H. Lie , G. A. Brooks , R. W. Davis , A. Javey , Nature 2016, 529, 509. 26819044 10.1038/nature16521 PMC4996079

[175] Y. Khan , M. Garg , Q. Gui , M. Schadt , A. Gaikwad , D. Han , N. A. D. Yamamoto , P. Hart , R. Welte , W. Wilson , S. Czarnecki , M. Poliks , Z. Jin , K. Ghose , F. Egitto , J. Turner , A. C. Arias , Adv. Funct. Mater. 2016, 26, 8764.

[176] Y. Liu , J. J. Norton , R. Qazi , Z. Zou , K. R. Ammann , H. Liu , L. Yan , P. L. Tran , K.‐I. Jang , J. W. Lee , Sci. Adv. 2016, 2, e1601185. 28138529 10.1126/sciadv.1601185 PMC5262452

[177] Y. Lee , C. Howe , S. Mishra , D. S. Lee , M. Mahmood , M. Piper , Y. Kim , K. Tieu , H. S. Byun , J. P. Coffey , M. Shayan , Y. Chun , R. M. Costanzo , W. H. Yeo , Proc. Natl. Acad. Sci. USA 2018, 115, 5377. 29735689 10.1073/pnas.1719573115 PMC6003521

[178] S. Wang , J. Xu , W. Wang , G.‐J. N. Wang , R. Rastak , F. Molina‐Lopez , J. W. Chung , S. Niu , V. R. Feig , J. Lopez , T. Lei , S.‐K. Kwon , Y. Kim , A. M. Foudeh , A. Ehrlich , A. Gasperini , Y. Yun , B. Murmann , J. B.‐H. Tok , Z. Bao , Nature 2018, 555, 83. 29466334 10.1038/nature25494

[179] D. Son , J. Lee , S. Qiao , R. Ghaffari , J. Kim , J. E. Lee , C. Song , S. J. Kim , D. J. Lee , S. W. Jun , S. Yang , M. Park , J. Shin , K. Do , M. Lee , K. Kang , C. S. Hwang , N. Lu , T. Hyeon , D.‐H. Kim , Nat. Nanotechnol. 2014, 9, 397. 24681776 10.1038/nnano.2014.38

[180] S. Imani , A. J. Bandodkar , A. M. V. Mohan , R. Kumar , S. Yu , J. Wang , P. P. Mercier , Nat. Commun. 2016, 7, 11650. 27212140 10.1038/ncomms11650 PMC4879240

[181] J. Kim , G. A. Salvatore , H. Araki , A. M. Chiarelli , Z. Xie , A. Banks , X. Sheng , Y. Liu , J. W. Lee , K.‐I. Jang , S. Y. Heo , K. Cho , H. Luo , B. Zimmerman , J. Kim , L. Yan , X. Feng , S. Xu , M. Fabiani , G. Gratton , Y. Huang , U. Paik , J. A. Rogers , Sci. Adv. 2016, 2, e1600418. 27493994 10.1126/sciadv.1600418 PMC4972468

[182] H. U. Chung , B. H. Kim , J. Y. Lee , J. Lee , Z. Xie , E. M. Ibler , K. Lee , A. Banks , J. Y. Jeong , J. Kim , C. Ogle , D. Grande , Y. Yu , H. Jang , P. Assem , D. Ryu , J. W. Kwak , M. Namkoong , J. B. Park , Y. Lee , D. H. Kim , A. Ryu , J. Jeong , K. You , B. Ji , Z. Liu , Q. Huo , X. Feng , Y. Deng , Y. Xu , K.‐I. Jang , J. Kim , Y. Zhang , R. Ghaffari , C. M. Rand , M. Schau , A. Hamvas , D. E. Weese‐Mayer , Y. Huang , S. M. Lee , C. H. Lee , N. R. Shanbhag , A. S. Paller , S. Xu , J. A. Rogers , Science 2019, 363, eaau0780.30819934

[183] M. K. Choi , O. K. Park , C. Choi , S. Qiao , R. Ghaffari , J. Kim , D. J. Lee , M. Kim , W. Hyun , S. J. Kim , Adv. Healthcare Mater. 2016, 5, 80. 10.1002/adhm.201500285 25989744

[184] M. B. I. Reaz , M. S. Hussain , F. Mohd‐Yasin , Biol. Proced. Online 2006, 8, 11. 19565309 10.1251/bpo124 PMC1622762

[185] K. I. Jang , H. N. Jung , J. W. Lee , S. Xu , Y. H. Liu , Y. J. Ma , J. W. Jeong , Y. M. Song , J. Kim , B. H. Kim , A. Banks , J. W. Kwak , Y. Y. Yang , D. W. Shi , Z. J. Wei , X. Feng , U. Paik , Y. G. Huang , R. Ghaffari , J. A. Rogers , Adv. Funct. Mater. 2016, 26, 7281. 28413376 10.1002/adfm.201603146 PMC5390688

[186] L. Rofes , N. Vilardell , P. Clavé , Neurogastroenterol. Motil. 2013, 25, 278. 23480388 10.1111/nmo.12112

[187] K. A. Hutcheson , J. S. Lewin , D. A. Barringer , A. Lisec , G. B. Gunn , M. W. Moore , F. C. Holsinger , Cancer 2012, 118, 5793. 23640737 10.1002/cncr.27631 PMC4034519

[188] G. Constantinescu , J. W. Jeong , X. D. Li , D. K. Scott , K. I. Jang , H. J. Chung , J. A. Rogers , J. Rieger , Med. Eng. Phys. 2016, 38, 807. 27255865 10.1016/j.medengphy.2016.04.023

[189] M. K. Kim , C. Kantarcigil , B. Kim , R. K. Baruah , S. Maity , Y. Park , K. Kim , S. Lee , J. B. Malandraki , S. Avlani , Sci. Adv. 2019, 5, eaay3210. 31853500 10.1126/sciadv.aay3210 PMC6910838

[190] T. Azim , M. A. Jaffar , A. M. Mirza , Appl. Soft Comput. 2014, 18, 25.

[191] F. Khosrow‐Khavar , K. Tavakolian , A. P. Blaber , J. M. Zanetti , R. Fazel‐Rezai , C. Menon , IEEE J. Biomed. Health Inf. 2014, 19, 1428. 10.1109/JBHI.2014.2360156 25265620

[192] O. T. Inan , P.‐F. Migeotte , K.‐S. Park , M. Etemadi , K. Tavakolian , R. Casanella , J. Zanetti , J. Tank , I. Funtova , G. K. Prisk , M. D. Rienz , IEEE J. Biomed. Health Inf. 2014, 19, 1414. 10.1109/JBHI.2014.2361732 25312966

[193] P. Li , B. Zhang , T. Cui , Biosens. Bioelectron. 2015, 72, 168. 25982724 10.1016/j.bios.2015.05.007

[194] S. K. Ameri , M. Kim , I. A. Kuang , W. K. Perera , M. Alshiekh , H. Jeong , U. Topcu , D. Akinwande , N. Lu , NPJ 2D Mater. Appl. 2018, 2, 19.

[195] S. Mishra , J. J. Norton , Y. Lee , D. S. Lee , N. Agee , Y. Chen , Y. Chun , W. H. Yeo , Biosens. Bioelectron. 2017, 91, 796. 28152485 10.1016/j.bios.2017.01.044 PMC5323068

[196] C. Babiloni , C. Del Percio , L. Arendt‐Nielsen , A. Soricelli , G. L. Romani , P. M. Rossini , P. Capotosto , Clin. Neurophysiol. 2014, 125, 1936. 24929901 10.1016/j.clinph.2014.04.021

[197] M. Mahmood , D. Mzurikwao , Y.‐S. Kim , Y. Lee , S. Mishra , R. Herbert , A. Duarte , C. S. Ang , W.‐H. Yeo , Nat. Mach. Intell. 2019, 1, 412.

[198] H. Yuk , B. Y. Lu , X. H. Zhao , Chem. Soc. Rev. 2019, 48, 1642. 30474663 10.1039/c8cs00595h

[199] B. Taji , S. Shirmohammadi , V. Groza , I. Batkin , IEEE Trans. Instrum. Meas. 2014, 63, 1412.

[200] S. Yao , A. Myers , A. Malhotra , F. Lin , A. Bozkurt , J. F. Muth , Y. Zhu , Adv. Healthcare Mater. 2017, 6, 1601159. 10.1002/adhm.201601159 28128888

[201] H. Wang , H. Wang , Y. Wang , X. Su , C. Wang , M. Zhang , M. Jian , K. Xia , X. Liang , H. Lu , S. Li , Y. Zhang , ACS Nano 2020, 14, 3219. 32083839 10.1021/acsnano.9b08638

[202] M. Jo , K. Min , B. Roy , S. Kim , S. Lee , J.‐Y. Park , S. Kim , ACS Nano 2018, 12, 5637. 29792681 10.1021/acsnano.8b01435

[203] H. Liu , W. Dong , Y. Li , F. Li , J. Geng , M. Zhu , T. Chen , H. Zhang , L. Sun , C. Lee , Microsyst. Nanoeng. 2020, 6, 16. 10.1038/s41378-019-0127-5 PMC8433406 34567631

[204] B. M. Li , I. Kim , Y. Zhou , A. C. Mills , T. J. Flewwellin , J. S. Jur , Adv. Mater. Technol. 2019, 4, 1900511.

[205] Y. Qiao , Y. Wang , J. Jian , M. Li , G. Jiang , X. Li , G. Deng , S. Ji , Y. Wei , Y. Pang , Carbon 2020, 156, 253.

[206] C. Yang , H. Zhang , Y. Liu , Z. Yu , X. Wei , Y. Hu , Adv. Sci. 2018, 5, 1801070. 10.1002/advs.201801070 PMC6299731 30581706

[207] T. G. La , S. Qiu , D. K. Scott , R. Bakhtiari , J. W. P. Kuziek , K. E. Mathewson , J. Rieger , H. J. Chung , Adv. Healthcare Mater. 2018, 7, 1801033. 10.1002/adhm.201801033 30338670

[208] G. J. Jin , M. J. Uddin , J. S. Shim , Adv. Funct. Mater. 2018, 28, 1804351.

[209] A. Achilli , A. Bonfiglio , D. Pani , IEEE Sens. J. 2018, 18, 4097.

[210] H. W. Kim , T. Y. Kim , H. K. Park , I. You , J. Kwak , J. C. Kim , H. Hwang , H. S. Kim , U. Jeong , ACS Appl. Mater. Interfaces 2018, 10, 40141. 30360058 10.1021/acsami.8b13857

[211] I. del Agua , D. Mantione , U. Ismailov , A. Sanchez‐Sanchez , N. Aramburu , G. G. Malliaras , D. Mecerreyes , E. Ismailova , Adv. Mater. Technol. 2018, 3, 1700322.

[212] H. Jin , M. O. G. Nayeem , S. Lee , N. Matsuhisa , D. Inoue , T. Yokota , D. Hashizume , T. Someya , ACS Nano 2019, 13, 7905. 31244040 10.1021/acsnano.9b02297

[213] D. Miao , Z. Huang , X. Wang , J. Yu , B. Ding , Small 2018, 14, 1801527. 10.1002/smll.201801527 30004631

[214] Y. M. Chi , G. Cauwenberghs , in Proc. 2010 Int. Conf. on Body Sensor Network, IEEE, Piscataway, NJ 2010, p. 297.

[215] E. Nemati , M. J. Deen , T. Mondal , IEEE Commun. Mag. 2012, 50, 36.

[216] Y. Li , Y. Luo , S. Nayak , Z. Liu , O. Chichvarina , E. Zamburg , X. Zhang , Y. Liu , C. H. Heng , A. V.‐Y. Thean , Adv. Electron. Mater. 2019, 5, 1800463.

[217] G. Yao , L. Xu , X. Cheng , Y. Li , X. Huang , W. Guo , S. Liu , Z. L. Wang , H. Wu , Adv. Funct. Mater. 2020, 30, 1907312.

[218] C. Zhang , S. Liu , X. Huang , W. Guo , Y. Li , H. Wu , Nano Energy 2019, 62, 164.

[219] Z. S. Wu , K. Parvez , X. L. Feng , K. Mullen , Nat. Commun. 2013, 4, 2487. 24042088 10.1038/ncomms3487 PMC3778542

[220] X. Peng , L. Peng , C. Wu , Y. Xie , Chem. Soc. Rev. 2014, 43, 3303. 24614864 10.1039/c3cs60407a

[221] A. Kurs , A. Karalis , R. Moffatt , J. D. Joannopoulos , P. Fisher , M. Soljačić , Science 2007, 317, 83. 17556549 10.1126/science.1143254

[222] Y. Yang , W. Gao , Chem. Soc. Rev. 2019, 48, 1465. 29611861 10.1039/c7cs00730b

[223] R. You , Y.‐Q. Liu , Y.‐L. Hao , D.‐D. Han , Y.‐L. Zhang , Z. You , Adv. Mater. 2020, 32, 1901981.

[224] H. Chae , H.‐J. Kwon , Y.‐K. Kim , Y. Won , D. Kim , H.‐J. Park , S. Kim , S. Gandla , ACS Appl. Mater. Interfaces 2019, 11, 28387. 31294964 10.1021/acsami.9b06363

[225] L. M. Ferrari , S. Sudha , S. Tarantino , R. Esposti , F. Bolzoni , P. Cavallari , C. Cipriani , V. Mattoli , F. Greco , Adv. Sci. 2018, 5, 1700771. 10.1002/advs.201700771 PMC5867059 29593975

[226] N. Karim , S. Afroj , A. Malandraki , S. Butterworth , C. Beach , M. Rigout , K. S. Novoselov , A. J. Casson , S. G. Yeates , J. Mater. Chem. C 2017, 5, 11640.

[227] Y. Xu , Y. Zhu , F. Han , C. Luo , C. Wang , Adv. Energy Mater. 2015, 5, 1400753.

[228] A. A. Chlaihawi , B. B. Narakathu , S. Emamian , B. J. Bazuin , M. Z. Atashbar , Sens. Bio‐Sens. Res. 2018, 20, 9.

[229] Y. Wang , Y. Qiu , S. K. Ameri , H. Jang , Z. Dai , Y. Huang , N. Lu , npj Flex. Electron. 2018, 2, 6.

[230] S. P. Lee , G. Ha , D. E. Wright , Y. J. Ma , E. Sen‐Gupta , N. R. Haubrich , P. C. Branche , W. H. Li , G. L. Huppert , M. Johnson , H. B. Mutlu , K. Li , N. Sheth , J. A. Wright , Y. G. Huang , M. Mansour , J. A. Rogers , R. Ghaffari , NPJ Digital Med. 2018, 1, 2. 10.1038/s41746-017-0009-x PMC6550217 31304288

[231] S. Debener , R. Emkes , M. De Vos , M. Bleichner , Sci. Rep. 2015, 5, 16743. 26572314 10.1038/srep16743 PMC4648079

[232] L. Inzelberg , D. Rand , S. Steinberg , M. David‐Pur , Y. Hanein , Sci. Rep. 2018, 8, 2058. 29391503 10.1038/s41598-018-20567-y PMC5794977

